# A Procedure for Deriving Formulas to Convert Transition Rates to Probabilities for Multistate Markov Models

**DOI:** 10.1177/0272989X17696997

**Published:** 2017-04-05

**Authors:** Edmund Jones, David Epstein, Leticia García-Mochón

**Affiliations:** University of Cambridge, Cambridge, England, UK (EJ); University of Granada, Granada, Spain (DE); Escuela Andaluza de Salud Pública, Granada, Spain (LG); Instituto de Investigación Biosanitaria ibs. Granada (LG)

**Keywords:** Markov model, transition rates, transition probabilities, spreadsheet

## Abstract

For health-economic analyses that use multistate Markov models, it is often necessary to convert from transition rates to transition probabilities, and for probabilistic sensitivity analysis and other purposes it is useful to have explicit algebraic formulas for these conversions, to avoid having to resort to numerical methods. However, if there are four or more states then the formulas can be extremely complicated. These calculations can be made using packages such as R, but many analysts and other stakeholders still prefer to use spreadsheets for these decision models. We describe a procedure for deriving formulas that use intermediate variables so that each individual formula is reasonably simple. Once the formulas have been derived, the calculations can be performed in Excel or similar software. The procedure is illustrated by several examples and we discuss how to use a computer algebra system to assist with it. The procedure works in a wide variety of scenarios but cannot be employed when there are several backward transitions and the characteristic equation has no algebraic solution, or when the eigenvalues of the transition rate matrix are very close to each other.

In discrete-time Markov chains, transitions are described in terms of probabilities, which represent the expected proportions that make the various transitions in each cycle or time-period. In continuous-time Markov chains, transitions are described in terms of rates, which represent the instantaneous incidences of transitions from one state to another. Medical decision models are commonly constructed in the form of multistate Markov models, and they are usually analyzed using discrete time-periods because this is more practical in spreadsheets and similar software. These models therefore require a set of transition probabilities as input.

From some primary data, it is possible to estimate transition probabilities directly.^1^ But it is common to estimate transition rates instead, mainly because relative rates from other sources, such as randomized controlled trials, can easily be incorporated into rate estimates using the assumption of proportional hazards. Methods for estimating transition rates in multistate settings with censoring and competing risks have been described elsewhere.^2^

It is then necessary to convert from transition rates to transition probabilities. It is common to use the formula $p\left(t\right)=1-e^{-\mathrm{rt}}$, where r is the rate and t is the cycle length (in this paper we refer to this as the “simple formula”). But this is incorrect for most models with two or more transitions, essentially because a person can experience more than one type of event in a single cycle. For example, they might go from healthy to ill and from ill to dead within a single cycle, or straight from healthy to dead. The simple formula is always wrong if there are competing risks (that is, if from one state there are two or more other states that a person can move to).

If the cycles are shortened then the simple formula will be more accurate, because a person is less likely to have two events in a single cycle. But this has several disadvantages. It increases the number of rows in the Excel spreadsheet, making the whole exercise more cumbersome; there is no simple answer to what lengths of cycles should be used to achieve an appropriate degree of accuracy; and of course it is mathematically incorrect, and correct methods are preferable. A further issue is that shorter cycles increase the computation time, though this would not be a big problem with modern computers and small models. (The simple formula is discussed again at the end of the paper.)

The paper illustrates the steps required to solve the Kolmogorov equations using the diagonalization approach. We show how to derive and apply algebraic formulas for the conversions that can be used for a wide variety of models with four or five states and some models with six or more states. The formulas use several sets of intermediate variables, so that the individual formulas are relatively simple and can be entered into an Excel spreadsheet, with one formula in each cell and no need for macros or Visual Basic. The mathematical methods themselves are not novel; the use of intermediate equations is really just a way of keeping the calculations tidy, and provides clarity for those who may not be familiar with the underlying mathematics.

Previous publications have given formulas for converting rates to probabilities for certain two- and three-state models^3-5^ and one four-state model.^4^ For most models with larger numbers of states, the formulas are extremely complicated and these publications recommend numerical methods with software such as R (the msm package) or WinBUGS and WBDiff. This approach may be practical if one is willing and able to implement the entire model in R or WinBUGS—though many analysts are still more comfortable working with Excel, for example because they consider it easier or because it facilitates presentation of results to other stakeholders. In principle, WinBUGS can convert the rates to probabilities and produce samples from a probabilistic sensitivity analysis, which can then be saved and copied into a spreadsheet containing the decision model. However, this procedure might be unwieldy as it involves multiple software packages. Moreover, it is common for transition rates to vary according to an external measure of time, such as the age of the patient, which means that if numerical methods are used then the conversion from rates to probabilities has to be done for each age-group or cycle separately.

Our approach is aimed at analysts who develop decision models in spreadsheets, and should also be of interest to analysts who want to understand the mathematical derivation of the formulas used to convert rates to probabilities. The idea is that the analyst can set up the formulas once, and then copy them so that they are used for each age-group or cycle. Univariate sensitivity analysis and probabilistic sensitivity analysis are straightforward, as the direct connection from the rates and their standard errors to the transition probabilities is maintained and there is no need to copy and paste the samples from elsewhere. Another advantage is that this approach might be easier to audit and validate than an analysis where several software packages are used.

First we describe the mathematical background. Then we describe the usual procedure for deriving formulas to convert transition rates to probabilities, for models with forward and backward transitions. We illustrate this for a three-state model. We then discuss the situations in which the procedure does not work (it is easier to explain these after an example). Next we describe the new procedure with intermediate variables and illustrate it by examples with four- and five-state models. We also discuss how to derive the formulas using a computer algebra system instead of pen and paper. The final section is a discussion.

For all our example models, the formulas are set out in the accompanying Excel files (see supplementary material). For state-transition models of the appropriate structures, these files can be used directly. The analyst can simply copy the formulas into their own Excel files or copy their own transition rates into a copy of one of our Excel files. For models with other structures, the appropriate formulas will have to be derived using our procedure.

## Kolmogorov’s Equations and the Matrix Exponential

Given the transition-rate matrix Q for a continuous-time Markov chain X with n states, the task is to calculate the n×n transition-probability matrix P(t), whose elements are $p_{\mathrm{ij}}\left(t\right)=P\left(X\left(t\right)=j|X\left(0\right)=i\right)$. P(t) is the solution to Kolmogorov’s forward and backward equations, P′(t)=P(t)Q and P′(t)=QP(t), with the initial condition that P(0) is the identity matrix I.^6,7^ Here P′(t) is the matrix whose (i,j)th element is $p_{\mathrm{ij}}\prime \left(t\right)=\frac{d}{\mathrm{dt}}p_{\mathrm{ij}}\left(t\right)$, so either of these matrix equations could alternatively be written as a set of $n^{2}$ scalar equations, one for each $p_{\mathrm{ij}}\prime \left(t\right)$.

In this paper we assume that n is finite, Q is constant, and in P(t) the row-sums are all 1 and in Q they are all 0 (that is, $\sum_{j=1}^{n}p_{\mathrm{ij}}\left(t\right)=1$ and $\sum_{j=1}^{n}q_{\mathrm{ij}}=0$ for i=1,…,n). With these assumptions, the forward and backward equations both have a unique solution:

$$P\left(t\right)=\mathrm{Exp}\left(\mathrm{Qt}\right)=\sum_{r=0}^{\infty }Q^{r}\frac{t^{r}}{r!}.$$

Here, Exp is the matrix exponential, which is defined by the infinite sum. This infinite sum is known to converge (see section 4.5(iii) of Cox and Miller^6^). In a strict mathematical sense, our assumptions about n, Q, and P(t) are more restrictive than they need to be—even if they were slightly relaxed, the matrix exponential would still be the unique solution to Kolmogorov’s equations—but the exact necessary and sufficient conditions are formidably complicated. Other publications provide full explanations^8,9^ and shorter accounts.^6,7^

If Q has an eigen-decomposition, then the matrix exponential can be expressed in a simple form. Let D be a diagonal matrix of eigenvalues of Q and let U be a corresponding matrix of eigenvectors, so that $Q=\mathrm{UD}U^{-1}$ and $\mathrm{Qt}=U\;\mathrm{Dt}\;U^{-1}$. It follows that

$$\mathrm{Exp}\left(\mathrm{Qt}\right)=U\;\mathrm{Exp}\left(\mathrm{Dt}\right)U^{-1}.$$

This is much simpler, since Exp(Dt) is a diagonal matrix whose (i,i)th element is simply $e^{d_{\mathrm{ii}}t}$.

Our formulas for the transition probabilities are based on this second formula for Exp(Qt) and assume that Q has an eigen-decomposition, which is usually the case. What happens when Q does not have an eigen-decomposition is discussed in the section after next.

There are several alternative terms and notations. Eigen-decomposition is sometimes known as spectral decomposition. If a matrix has an eigen-decomposition then it is said to be diagonalizable. Q is sometimes called the generator matrix and written as G. It can be convenient to write $q_{i}$ instead of $-q_{\mathrm{ii}}$, so that $q_{i}=\sum_{i\neq j}q_{\mathrm{ij}}\geq 0$.^8^

## A Procedure for Deriving the Formulas When *n* is Small

For a given multistate Markov model, the formulas for $p_{\mathrm{ij}}\left(t\right)$ in terms of $q_{\mathrm{ij}}$ can be derived by carrying out the following steps:

Step 1. Write down Q, with algebraic symbols like $q_{12}$ for transitions that are allowed and zeroes for transitions that are not allowed.
Step 2. Derive formulas for the elements of D by solving the characteristic equation det(Q−λI)=0 (the diagonal elements of D are the values of λ that solve this equation).
Step 3. Derive formulas for the elements of U by solving Qx=λx, where x is an n×1 vector (the columns of U are the values of x that solve this equation).
Step 4. Derive formulas for the elements of $U^{-1}$ by any of several standard methods (for example, using the matrix of cofactors).
Step 5. Derive formulas for the elements of $P\left(t\right)=U\;\mathrm{Exp}\left(\mathrm{Dt}\right)U^{-1}$ by using the rules of matrix multiplication.

Our presentation of this procedure is novel but mathematically these steps are closely based on the results in the previous section.

In Steps 2 and 3, the diagonal elements of D and the columns of U do not have to be in any particular order, but they must match each other so that the eigenvalue $d_{\mathrm{ii}}$ corresponds to the ith column of U.

At each step, the formulas should be simplified using the standard rules of algebra. There are also several other ways of simplifying the formulas. Firstly, if the only possible transition from i is from i to j, then $q_{\mathrm{ii}}$ can be replaced by $-q_{\mathrm{ij}}$ in Step 1. (If more than one transition from i is possible then $q_{\mathrm{ii}}$ can be replaced by $-\sum_{j\neq i}q_{\mathrm{ij}}$, though this often makes the formulas more complicated.) Secondly, if x is an eigenvector of Q with eigenvalue λ then the same is true of ax, for any a∈R, so in Step 3 the formulas can be simplified by multiplying the columns of U by scalars. Thirdly, each row of P(t) must sum to 1, so in Step 5 $p_{\mathrm{ij}}\left(t\right)$ can be replaced by $1-\sum_{k\neq j}p_{\mathrm{ik}}\left(t\right)$. This will mean that the formula for $p_{\mathrm{ij}}\left(t\right)$ is not in terms of elements of Q directly but in terms of other elements of P(t)—obviously this can only be done for one element in each row of P(t).

If there are only forward transitions, then Step 2 is simple, because Q is an upper-triangular matrix and the values of λ are just the diagonal elements of Q. If there are backward transitions then the characteristic equation can often be solved by noticing that the left-hand side has certain factors or using the well-known formula for the solutions of a quadratic equation. Otherwise it may be necessary to use the formulas for the solutions of cubic or quartic equations,^10–12^ though these are complicated and usually written in terms of intermediate variables themselves. If it is impractical or impossible to solve the characteristic equation, then the procedure will not work, as discussed in the next section.

The following is an illustration of the procedure.

### Model 1. Three-State Model with Forward Transitions Only (See Figure 1)

Step 1: $Q=\left(\begin{array}{ccc} q_{11} & q_{12} & q_{13}\\ 0 & -q_{23} & q_{23}\\ 0 & 0 & 0 \end{array}\right)$, where $q_{11}=-q_{12}-q_{13}$
Step 2: $D=\left(\begin{array}{ccc} q_{11} & 0 & 0\\ 0 & -q_{23} & 0\\ 0 & 0 & 0 \end{array}\right)$
Step 3: $U=\left(\begin{array}{ccc} 1 & q_{12} & 1\\ 0 & -q_{11}-q_{23} & 1\\ 0 & 0 & 1 \end{array}\right)$
Step 4: $U^{-1}=\left(\begin{array}{ccc} 1 & \frac{q_{12}}{q_{11}+q_{23}} & \frac{q_{13}-q_{23}}{q_{11}+q_{23}}\\ 0 & -\frac{1}{q_{11}+q_{23}} & \frac{1}{q_{11}+q_{23}}\\ 0 & 0 & 1 \end{array}\right)$
Step 5: $P\left(t\right)=\left(\begin{array}{ccc} 1 & q_{12} & 1\\ 0 & -q_{11}-q_{23} & 1\\ 0 & 0 & 1 \end{array}\right)\;\left(\begin{array}{ccc} e^{q_{11}t}\; & 0 & 0\\ 0 & e^{-q_{23}t} & 0\\ 0 & 0 & 1 \end{array}\right)\;\left(\begin{array}{ccc} 1 & \frac{q_{12}}{q_{11}+q_{23}} & \frac{q_{13}-q_{23}}{q_{11}+q_{23}}\\ 0 & -\frac{1}{q_{11}+q_{23}} & \frac{1}{q_{11}+q_{23}}\\ 0 & 0 & 1 \end{array}\right)$

The final matrix here is equivalent to the final matrix in Figure 3 of Welton and Ades.^3^ Formulas for the three-state model with all forward transitions and the backward transition from state 2 to state 1 have also been published.^4^

**Figure 1:**
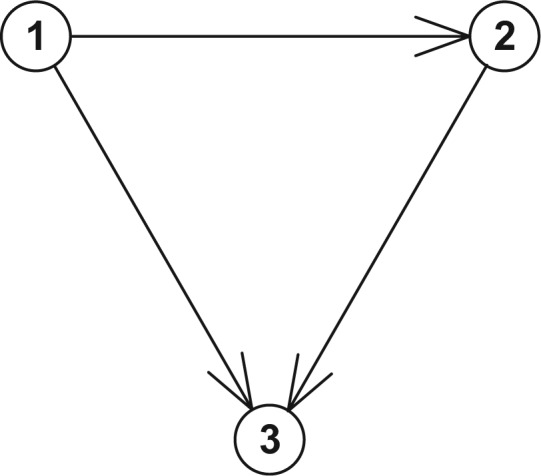
The states and transitions for Model 1, a three-state model with forward transitions only.

## Situations Where the Procedure Fails

For some models with five or more states, especially ones with several backward transitions, it is impossible to find an algebraic solution of the characteristic equation (this follows from the Abel–Ruffini theorem^13^). Step 2 therefore fails. If this happens, it will be necessary to use the numerical methods mentioned in the introduction or calculate the matrix exponential Exp(Qt) by other means. Calculating the matrix exponential in a reliable way is fundamentally difficult,^14,15^ but improved methods have appeared in recent years.^16,17^ Functions are available in Matlab, R, and Python. For R, see the expm package,^18^ the MatrixExp function in the msm package,^19^ or the msm vignette.^5^ The matrix exponential can even be calculated in Excel,^20^ but this requires purchasing an extra software library to run in the background.

If the characteristic equation has an algebraic solution, then our method will usually work. But if two eigenvalues turn out to be exactly equal when numbers are put into the formulas, then it will fail. This is rare, because the rates are numbers on the continuous real line and it is unlikely that, for example, two of them will be exactly equal. But if it happens, then attempting to use the formulas will result in division by zero and the software will raise an error or give an output of infinity. This can happen either in Step 3, if the formulas for the eigenvectors involve division, or in Step 4, when the matrix is inverted.

Problems can also arise if two of the eigenvalues are very close to each other, or if certain other numbers are very close; if the difference of two such numbers appears in a denominator, then the result can be inaccurate (which other numbers this applies to depends on the model and how the formulas are written; for example, in Model 3 v=bf−ag and the problem arises if this is close to zero). There is no clear-cut rule for whether two numbers are too close, but most software stores non-integer numbers in double-precision floating point format,^21,22^ which means about 16 significant figures, so roughly speaking there might be a problem if the numbers are the same to more than 8 or 10 significant figures. The problem of two eigenvalues being very close is more likely to arise in a PSA, so in a PSA it might be worth making scatter plots of the intermediate variables to see if any have extreme values, and possibly discarding those if there are only a few of them.

Lastly, if the model is beyond a certain size, then solving the characteristic equation algebraically may be possible in theory but too complicated in practice. These issues mean that output from the formulas should always be treated with caution. If the probabilities seem implausible then it will be necessary to calculate the matrix exponential by other methods as described above. For PSA it may also be worth making scatter plots of the final probabilities to check that they look plausible.

In some models the eigenvalues might be complex—that is, one or more of them involves the square root of a negative number. If this happens in Excel then there will be a #NUM! error, and the formulas will need to be rewritten using functions such as IMSQRT and IMSUM, but the procedure should still work. In our five example models, the eigenvectors are all always real.

## A Procedure for Larger *n*, Using Intermediate Variables

In theory, the procedure described above works for any n, so long as formulas can be found for the eigenvalues in Step 2. But if n is greater than 4 or so then the final formulas for the elements of P(t) are extremely long and complicated, even after they are simplified. So instead it is preferable to use three sets of intermediate variables. The first set of intermediate variables corresponds to the elements of D, the second to the elements of U, and the third to the elements of $U^{-1}$. The procedure is best explained by examples, and three examples are given below.

### Model 2. Four-State Model with Forward Transitions Only (See Figure 2)

Our work on this procedure arose from an empirical application for which this four-state model can be used. The four states are “healthy,”“had minor cardiovascular event,”“had major cardiovascular event,” and “dead.” The reason for having two cardiovascular disease states is that when a person has had a minor event they are more likely to go on to have a major event, and the mortality rate after a major event is greater than the mortality rate after a minor event.

**Figure 2:**
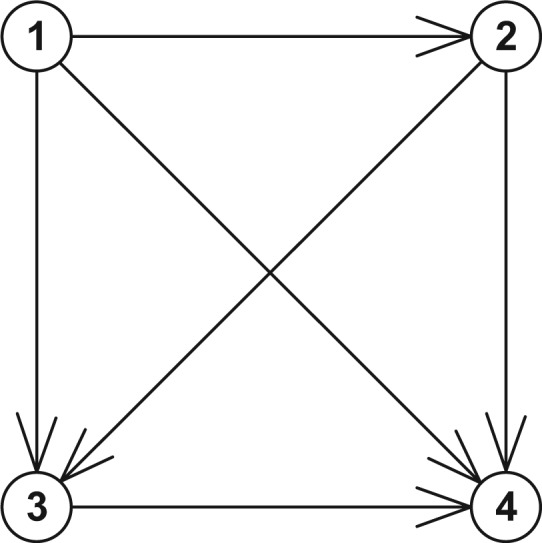
The states and transitions for Model 2, a four-state model with forward transitions only.

Single roman letters like a are used, since these are easier to read.

Step 1: $Q=\left(\begin{array}{cccc} a & b & c & d\\ 0 & f & g & h\\ 0 & 0 & -i & i\\ 0 & 0 & 0 & 0 \end{array}\right)$, where a=−b−c−d, and f=−g−h.
Step 2: Q is upper-triangular and so D just uses the values from the diagonal of Q.
  $$D=\left(\begin{array}{cccc} a & 0 & 0 & 0\\ 0 & f & 0 & 0\\ 0 & 0 & -i & 0\\ 0 & 0 & 0 & 0 \end{array}\right)$$
  For this model, there is no need to introduce intermediate variables at this step.
Step 3: In this step, U is worked out and then each element of U is written as a new intermediate variable, unless it is already a fixed number (such as 0 or 1) or a single letter (such as b in this example).
  As an illustration, the second column of U can be worked out as follows. Call this column β. This satisfies Qβ=fβ, or (Q−fI)β=0, so its elements can be found by writing either of these out as four simultaneous scalar equations (for example $\left(a-f\right)\beta_{1}+b\beta_{2}+c\beta_{3}+d\beta_{4}=0$, $g\beta_{3}+h\beta_{4}=0$, $\left(-i-f\right)\beta_{3}+i\beta_{4}=0$, and $-f\beta_{4}=0$), solving these, and multiplying by a factor to make the formulas simpler. There are any number of solutions but they all satisfy $\beta_{2}=\beta_{1}\left(f-a\right)/b$ and $\beta_{3}=\beta_{4}=0$.
  $$U=\left(\begin{array}{cccc} 1 & b & ci-bg+cf & bg+bh-cf-df\\ 0 & f-a & ag+gi & -ag-ah\\ 0 & 0 & -i^{2}-ai-af-fi & af\\ 0 & 0 & 0 & af \end{array}\right)=\left(\begin{array}{cccc} 1 & b & j & k\\ 0 & l & m & n\\ 0 & 0 & o & p\\ 0 & 0 & 0 & p \end{array}\right)$$
  The intermediate variables are j, k, l, and so on. The formulas for the intermediate variables can be read off: j=ci−bg+cf, k=bg+bh−cf−df, and so on.
Step 4: In this step, $U^{-1}$ is worked out, and then each element of $U^{-1}$ is written as a new intermediate variable, unless it is already a fixed number or a single letter.
  As an illustration, the (1,2) element of $U^{-1}$ can be worked out as follows. If C is the cofactor matrix of U, then $c_{21}$ is ${\left(-1\right)}^{2+1}\mathrm{bop}=-\mathrm{bop}$, and detU is simply lop (this is most easily found by expanding along the first column of U). So $u_{12}^{-1}=c_{21}/\det U=-b/l$.
  $$U^{-1}=\left(\begin{array}{cccc} 1 & -\frac{b}{l} & \frac{\mathrm{bm}}{\mathrm{lo}}-\frac{j}{o} & \frac{\mathrm{bn}}{\mathrm{lp}}-\frac{k}{p}-\frac{\mathrm{bm}}{\mathrm{lo}}+\frac{j}{o}\\ 0 & \frac{1}{l} & -\frac{m}{\mathrm{lo}} & \frac{m}{\mathrm{lo}}-\frac{n}{\mathrm{lp}}\\ 0 & 0 & \frac{1}{o} & -\frac{1}{o}\\ 0 & 0 & 0 & \frac{1}{p} \end{array}\right)=\left(\begin{array}{cccc} 1 & q & r & s\\ 0 & u & v & w\\ 0 & 0 & x & y\\ 0 & 0 & 0 & z \end{array}\right)$$
  The formulas for the new intermediate variables are $q=-\frac{b}{l}$, $r=\frac{\mathrm{bm}}{\mathrm{lo}}-\frac{j}{o}$, and so on.
Step 5: $\begin{array}{l} P\left(t\right)=Exp\left(Qt\right)=U\;Exp\left(Dt\right)\;U^{-1}\\ =\left(\begin{array}{cccc} e^{at} & bue^{ft}+qe^{at} & jxe^{-it}+bve^{ft}+re^{at} & kz+jye^{-it}+bwe^{ft}+se^{at}\\ 0 & lue^{ft} & mxe^{-it}+lve^{ft} & nz+mye^{-it}+lwe^{ft}\\ 0 & 0 & oxe^{-it} & pz+oye^{-it}\\ 0 & 0 & 0 & pz \end{array}\right). \end{array}$
The formulas for the transition probabilities are $p_{11}\left(t\right)=e^{\mathrm{at}}$, $p_{12}\left(t\right)=\mathrm{bu}e^{\mathrm{ft}}+qe^{\mathrm{at}}$, and so on. In general, if i is a death state (that is, an absorbing state) then $p_{\mathrm{ii}}\left(t\right)=1$. So, for this model,$p_{44}\left(t\right)$ is actually 1, and the pz in the formula for $p_{34}\left(t\right)$ can also be replaced by 1. For comparison, a single set of formulas for this model, with no intermediate variables, is given in section 3.3 of Welton.^4^ Because this model is reasonably simple, the single set of formulas is probably easier to use, but the formulas with intermediate variables are probably easier to derive.

### Model 3. Four-State Model with Forward Transitions and One Backward Transition (See Figure 3)

Step 1: $Q=\left(\begin{array}{cccc} a & b & c & d\\ f & g & h & i\\ 0 & 0 & -j & j\\ 0 & 0 & 0 & 0 \end{array}\right)$
Step 2: The characteristic equation is
  det(Q−λI)=0⇔(a−λ)(g−λ)(−j−λ)(−λ)−bf(−j−λ)(−λ)=0.
  The solutions λ=−j and λ=0 can be found by noticing that −j−λ and −λ are factors of the left-hand side. The other solutions can then be found by using the formula for the solutions of quadratic equations, and they can then be slightly simplified to $\left(a+g\pm \sqrt{{\left(a-g\right)}^{2}+4\mathrm{bf}}\right)/2$.
  $$\begin{array}{c} D=\left(\begin{array}{cccc} \frac{a+g+\sqrt{{\left(a-g\right)}^{2}+4\mathrm{bf}}}{2} & 0 & 0 & 0\\ 0 & \frac{a+g-\sqrt{{\left(a-g\right)}^{2}+4\mathrm{bf}}}{2} & 0 & 0\\ 0 & 0 & -j & 0\\ 0 & 0 & 0 & 0 \end{array}\right)=\left(\begin{array}{cccc} k & 0 & 0 & 0\\ 0 & l & 0 & 0\\ 0 & 0 & -j & 0\\ 0 & 0 & 0 & 0 \end{array}\right) \end{array}$$
  The intermediate variables here are k and l, and the formulas for them can be read off from the previous line. It would also be possible to replace −j with an intermediate variable.
Step 3:
  $$\begin{array}{c} U=\left(\begin{array}{cccc} 2b & 2b & \mathrm{cg}+\mathrm{cj}-\mathrm{bh} & \mathrm{cg}+\mathrm{dg}-\mathrm{bh}-\mathrm{bi}\\ -a+g+\sqrt{{\left(a-g\right)}^{2}+4\mathrm{bf}} & -a+g-\sqrt{{\left(a-g\right)}^{2}+4\mathrm{bf}} & \mathrm{ah}-\mathrm{cf}+\mathrm{hj} & \mathrm{ah}+\mathrm{ai}-\mathrm{cf}-\mathrm{df}\\ 0 & 0 & \mathrm{bf}-\mathrm{ag}-\mathrm{aj}-\mathrm{gj}-j^{2} & \mathrm{bf}-\mathrm{ag}\\ 0 & 0 & 0 & \mathrm{bf}-\mathrm{ag} \end{array}\right)\\ =\left(\begin{array}{cccc} m & m & n & o\\ p & q & r & s\\ 0 & 0 & u & v\\ 0 & 0 & 0 & v \end{array}\right) \end{array}$$
  This time $u_{11}$ and $u_{12}$ are the same, and so they can both be written as the same intermediate variable, m. The intermediate variables are m=2b, n=cg+cj−bh, and so on.
Step 4:
  $$U^{-1}=\left(\begin{array}{cccc} \frac{p}{\mathrm{mq}-\mathrm{mp}}+\frac{1}{m} & -\frac{1}{q-p} & -\frac{\mathrm{np}}{\mathrm{mqu}-\mathrm{mpu}}+\frac{r}{\mathrm{qu}-\mathrm{pu}}-\frac{n}{\mathrm{mu}} & -\frac{\mathrm{op}}{\mathrm{mqv}-\mathrm{mpv}}+\frac{s}{\mathrm{qv}-\mathrm{pv}}-\frac{o}{\mathrm{mv}}+\frac{\mathrm{np}}{\mathrm{mqu}-\mathrm{mpu}}-\frac{r}{\mathrm{qu}-\mathrm{pu}}+\frac{n}{\mathrm{mu}}\\ -\frac{p}{\mathrm{mq}-\mathrm{mp}} & \frac{1}{q-p} & \frac{\mathrm{np}}{\mathrm{mqu}-\mathrm{mpu}}-\frac{r}{\mathrm{qu}-\mathrm{pu}} & \frac{\mathrm{op}}{\mathrm{mqv}-\mathrm{mpv}}-\frac{s}{\mathrm{qv}-\mathrm{pv}}-\frac{\mathrm{np}}{\mathrm{mqu}-\mathrm{mpu}}+\frac{r}{\mathrm{qu}-\mathrm{pu}}\\ 0 & 0 & \frac{1}{u} & -\frac{1}{u}\\ 0 & 0 & 0 & \frac{1}{v} \end{array}\right)=\left(\begin{array}{cccc} a\prime & b\prime & c\prime & d\prime \\ f\prime & g\prime & h\prime & i\prime \\ 0 & 0 & j\prime & k\prime \\ 0 & 0 & 0 & l\prime \end{array}\right)$$
Step 5:
  $$P\left(t\right)=U\;\mathrm{Exp}\left(\mathrm{Dt}\right)U^{-1}=\left(\begin{array}{cccc} \mathrm{mf}\prime e^{\mathrm{lt}}+\mathrm{ma}\prime e^{\mathrm{kt}} & \mathrm{mg}\prime e^{\mathrm{lt}}+mb\prime e^{\mathrm{kt}} & \mathrm{nj}\prime e^{-\mathrm{jt}}+\mathrm{mh}\prime e^{\mathrm{lt}}+\mathrm{mc}\prime e^{\mathrm{kt}} & \mathrm{ol}\prime +\mathrm{nk}\prime e^{-\mathrm{jt}}+\mathrm{mi}\prime e^{\mathrm{lt}}+\mathrm{md}\prime e^{\mathrm{kt}}\\ \mathrm{qf}\prime e^{\mathrm{lt}}+\mathrm{pa}\prime e^{\mathrm{kt}} & \mathrm{qg}\prime e^{\mathrm{lt}}+\mathrm{pb}\prime e^{\mathrm{kt}} & \mathrm{rj}\prime e^{-\mathrm{jt}}+\mathrm{qh}\prime e^{\mathrm{lt}}+\mathrm{pc}\prime e^{\mathrm{kt}} & \mathrm{sl}\prime +\mathrm{rk}\prime e^{-\mathrm{jt}}+\mathrm{qi}\prime e^{\mathrm{lt}}+\mathrm{pd}\prime e^{\mathrm{kt}}\\ 0 & 0 & \mathrm{uj}\prime e^{-\mathrm{jt}} & 1+\mathrm{uk}\prime e^{-\mathrm{jt}}\\ 0 & 0 & 0 & 1 \end{array}\right)$$

**Figure 3:**
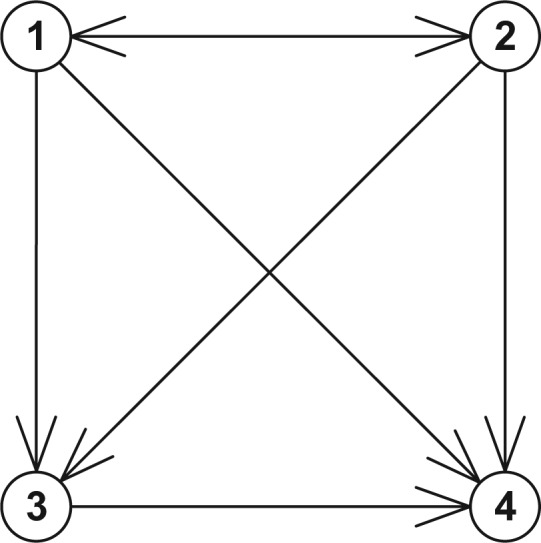
The states and transitions for Model 3, a four-state model with forward transitions and one backward transition.

### Model 4. Five-State Model with Forward Transitions Only and Two Death States (See Figure 4)

Step 1: $Q=\left(\begin{array}{ccccc} a & b & c & d & f\\ 0 & g & h & i & j\\ 0 & 0 & k & l & m\\ 0 & 0 & 0 & 0 & 0\\ 0 & 0 & 0 & 0 & 0 \end{array}\right)$
Step 2: $D=\left(\begin{array}{ccccc} a & 0 & 0 & 0 & 0\\ 0 & g & 0 & 0 & 0\\ 0 & 0 & k & 0 & 0\\ 0 & 0 & 0 & 0 & 0\\ 0 & 0 & 0 & 0 & 0 \end{array}\right)$
Step 3:
  $$\begin{array}{cc} U= & \left(\begin{array}{ccccc} 1 & b & \mathrm{bh}+\mathrm{ck}-\mathrm{cg} & \mathrm{cgl}-\mathrm{dgk}-\mathrm{bhl}+\mathrm{bik} & \mathrm{cgm}-\mathrm{fgk}-\mathrm{bhm}+\mathrm{bjk}\\ 0 & g-a & \mathrm{hk}-\mathrm{ah} & \mathrm{ahl}-\mathrm{aik} & \mathrm{ahm}-\mathrm{ajk}\\ 0 & 0 & k^{2}-\mathrm{gk}-\mathrm{ak}+\mathrm{ag} & -\mathrm{agl} & -\mathrm{agm}\\ 0 & 0 & 0 & \mathrm{agk} & 0\\ 0 & 0 & 0 & 0 & \mathrm{agk} \end{array}\right)=\left(\begin{array}{ccccc} 1 & b & n & o & p\\ 0 & q & r & s & u\\ 0 & 0 & v & w & x\\ 0 & 0 & 0 & y & 0\\ 0 & 0 & 0 & 0 & y \end{array}\right) \end{array}$$
  The formulas for the intermediate variables in U are n=bh+ck−cg, o=cgl−dgk−bhl+bik, and so on.
Step 4:
  $$\begin{array}{c} U^{-1}=\left(\begin{array}{ccccc} 1 & -\frac{b}{q} & \frac{\mathrm{br}}{\mathrm{qv}}-\frac{n}{v} & -\frac{\mathrm{brw}}{\mathrm{qvy}}+\frac{\mathrm{nw}}{\mathrm{vy}}+\frac{\mathrm{bs}}{\mathrm{qy}}-\frac{o}{y} & -\frac{\mathrm{brx}}{\mathrm{qvy}}+\frac{\mathrm{nx}}{\mathrm{vy}}+\frac{\mathrm{bu}}{\mathrm{qy}}-\frac{p}{y}\\ 0 & \frac{1}{q} & -\frac{r}{\mathrm{qv}} & \frac{\mathrm{rw}}{\mathrm{qvy}}-\frac{s}{\mathrm{qy}} & \frac{\mathrm{rx}}{\mathrm{qvy}}-\frac{u}{\mathrm{qy}}\\ 0 & 0 & \frac{1}{v} & -\frac{w}{\mathrm{vy}} & -\frac{x}{\mathrm{vy}}\\ 0 & 0 & 0 & \frac{1}{y} & 0\\ 0 & 0 & 0 & 0 & \frac{1}{y} \end{array}\right)=\left(\begin{array}{ccccc} 1 & a\prime & b\prime & c\prime & d\prime \\ 0 & f\prime & g\prime & h\prime & i\prime \\ 0 & 0 & j\prime & k\prime & l\prime \\ 0 & 0 & 0 & m\prime & 0\\ 0 & 0 & 0 & 0 & m\prime \end{array}\right) \end{array}$$
  The formulas for the intermediate variables in $U^{-1}$ are $a\prime =-\frac{b}{q}$, $b^{\prime }=\frac{\mathrm{br}}{\mathrm{qv}}-\frac{n}{v}$, and so on.
Step 5:
$P\left(t\right)=U\;\mathrm{Exp}\left(\mathrm{Dt}\right)U^{-1}=\left(\begin{array}{ccccc} e^{\mathrm{at}} & \mathrm{bf}\prime e^{\mathrm{gt}}+a\prime e^{\mathrm{at}} & \mathrm{nj}\prime e^{\mathrm{kt}}+\mathrm{bg}\prime e^{\mathrm{gt}}+b\prime e^{\mathrm{at}} & \mathrm{om}\prime +\mathrm{nk}\prime e^{\mathrm{kt}}+\mathrm{bh}\prime e^{\mathrm{gt}}+c\prime e^{\mathrm{at}} & \mathrm{pm}\prime +\mathrm{nl}\prime e^{\mathrm{kt}}+\mathrm{bi}\prime e^{\mathrm{gt}}+d\prime e^{\mathrm{at}}\\ 0 & \mathrm{qf}\prime e^{\mathrm{gt}} & \mathrm{rj}\prime e^{\mathrm{kt}}+\mathrm{qg}\prime e^{\mathrm{gt}} & \mathrm{sm}\prime +\mathrm{rk}\prime e^{\mathrm{kt}}+\mathrm{qh}\prime e^{\mathrm{gt}} & \mathrm{um}\prime +\mathrm{rl}\prime e^{\mathrm{kt}}+\mathrm{qi}\prime e^{\mathrm{gt}}\\ 0 & 0 & \mathrm{vj}\prime e^{\mathrm{kt}} & \mathrm{wm}\prime +\mathrm{vk}\prime e^{\mathrm{kt}} & \mathrm{xm}\prime +\mathrm{vl}\prime e^{\mathrm{kt}}\\ 0 & 0 & 0 & 1 & 0\\ 0 & 0 & 0 & 0 & 1 \end{array}\right).$

**Figure 4:**
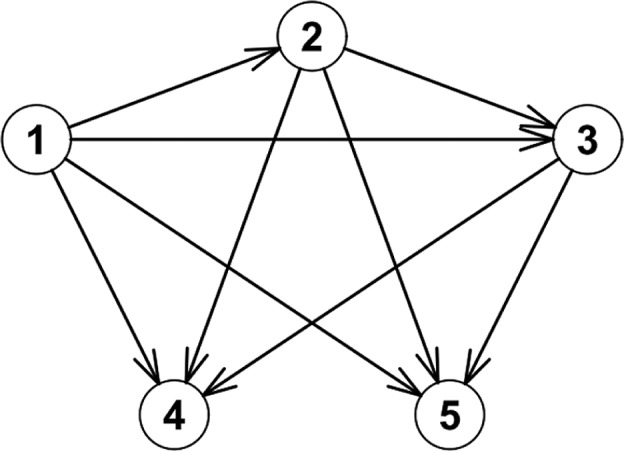
The states and transitions for Model 4, a five-state model with forward transitions only and two death states.

The formulas created by this procedure are suitable for working in Excel with one cell at a time. Each set of numbers or formulas can be arranged in the form of a matrix, and these matrices can be placed next to each other, for example in the order Q, D, U, $U^{-1}$, Exp(Dt), P(t). Alternatively, all the formulas can be put in a single row. The second way is probably more familiar and practical for a model that has different transition rates for each cycle or age-group. The supplementary material consists of Excel files that show how to lay out the formulas in both these ways, for the four example models. The files contain example numbers for the transition rates so that it is easy to see how the calculations are made.

The main reason why using intermediate variables is preferable to using a single set of direct formulas is that the formulas are much simpler. They are also easier to understand and organize because they correspond exactly to the matrices and equations for the solution to Kolmogorov’s equations. The disadvantage is that it involves more formulas.

The idea of using intermediate variables to simplify formulas for transition probabilities has been used in the field of applied biostatistics. See for example section 4.2 of Chiang,^23^ which is about models in which there are two alive states, with transitions both ways between them, and an arbitrary number of death states. Intermediate variables are used for the two non-zero eigenvalues.

## Using a Computer Algebra System

The four sets of formulas described in the previous sections are derived by eigen-decomposition, matrix inversion, and matrix multiplication. These derivations can all be done using pen and paper but it may be easier and more reliable to use a computer algebra system (CAS). The best-known CASs are Maple, Mathematica, and Matlab. In Matlab, the functions “eig” and “inv” can be used to find D, U, and $U^{-1}$. There is even a function called “expm” that can find the matrix exponential of a symbolic matrix (a matrix containing algebraic symbols like x). A single set of formulas for the transition probabilities could be read off from the output of this function. The disadvantages of this are firstly that, for models with four or more states the formulas will be extremely complicated and not practical for putting into Excel, and, secondly, that Matlab is not free or universally available.

There are various free CASs but not all of them have the necessary capabilities for deriving the formulas. One that does is Maxima.^24^ The code below shows what a user might type in Maxima to work out the formulas for Model 2 as shown above. The code is not a template that can be adapted to other models by simple changes like replacing zeroes with letters. Instead, after each step it is necessary to look at the output and decide what to type next. Maxima does not necessarily give the formulas in their simplest possible forms, so there is a need for judgement and trial and error in deciding how to define Q (for example, whether to use $-\sum_{j\neq 1}q_{1j}$ instead of $q_{11}$) and how to simplify the formulas. In Maxima, assignment is done using the colon, and lines must end with either a semi-colon, which tells the application to display the output, or a dollar symbol, which tells it not to.

If Step 2 involves solving a cubic or quartic equation, then that is likely to be slow even with a CAS, and of course if there are several backward transitions and no algebraic solution, then a CAS will not be able to get around this problem.

### Model 2. Four-State Model with Forward Transitions Only

/***** Step 1 *****/

/* Define Q. */

Q: matrix([a,b,c,d], [0,f,g,h], [0,0,-i,i], [0,0,0,0]);

/***** Step 2 *****/

/* Get the eigen-decomposition and reorder and display the eigenvalues. */

[evalues, evectors]: eigenvectors(Q);

evalues[1]: [evalues[1][3], evalues[1][2], evalues[1][1], evalues[1][4]];

/***** Step 3 *****/

/* Reorder and scale the eigenvectors, combine them to make U, and redefine U with intermediate variables. */

evectors: [evectors[3], evectors[2]*b, evectors[1]*(c*i-b*g+c*f), evectors[4]*(b*h+b*g+ (-d-c)*f)];

U: transpose(matrix(evectors[1][1]));

for i: 2 thru 4 do U: addcol(U, evectors[i][1]);

expand(U);

U: matrix([1,b,j,k], [0,l,m,n], [0,0,o,p], [0,0,0,p]);

/***** Step 4 *****/

/* Invert U and redefine Uinverse with intermediate values. */

expand(invert(U));

Uinverse: matrix([1,q,r,s], [0,u,v,w], [0,0,x,y], [0,0,0,z]);

/***** Step 5 *****/

/* Define expDt and find P(t). */

expDt: matrix([expat,0,0,0], [0,expft,0,0], [0,0,expminusit,0], [0,0,0,1]);

U . expDt . Uinverse;

Probably the easiest way to use Maxima is wxMaxima, which has a graphical interface. One setting that may be useful is: Edit – Configure – Enter evaluates cells. For simplifying, useful functions are “expand” and “ratsimp”.

## Discussion

For any given model, the four sets of formulas can be worked out by hand or by using a CAS, so long as the characteristic equation has an algebraic solution. Because the eigenvectors can be multiplied by constants, there are countless different possibilities for the formulas, but when the formulas are used on numerical transition rates, the results should be the same.

**Figure 5:**
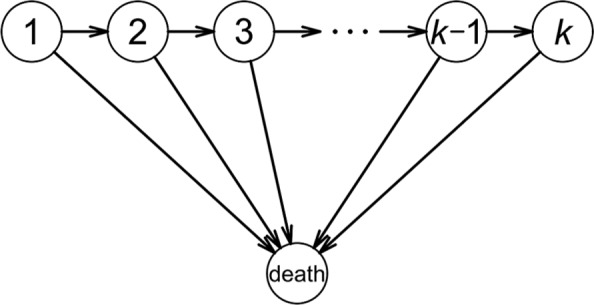
The disease progression model.

There is a rich variety of multistate Markov models with n=4, 5, or 6, and our procedure with intermediate variables works well with many of these. For n=4 or n=5 it is a matter of personal preference which methods are used to derive the formulas. For larger n, the formulas can become extremely complicated, but there are certain classes of models for which our procedure should still work. For example, the disease progression model (Figure 5) only allows patients to stay in the current state i, transition to the next state i+1, or die; there are no backward transitions. This type of model is relatively simple but not trivial and is often used, for example, in models of chronic progressive disease, or in cases where patients may undergo a predefined sequence of treatments. More generally, Markov models with n>4 are common, but the number of transitions from each state is often less than 4 or 5, so the algebra is not necessarily too complicated and our procedure should work in some of these situations.

An analyst who is familiar with R would probably prefer to develop the entire decision model in R, using a package such as expm to calculate the matrix exponential. However, many analysts still prefer to develop decision models in spreadsheets, and this paper is aimed at them. In the case where a decision model has been developed in a spreadsheet, the current alternative to our approach for calculating probabilities from rates would be to calculate the matrix exponential using an external package such as R or WBDiff and copy and paste the results into the spreadsheet.

An advantage of our algebraic formulas for the transition probabilities over numerical methods such as WBDiff is the speed and simplicity of running the calculations multiple times for probabilistic sensitivity analyses. PSA enables calculation of the overall probability that a treatment is more effective or cost-effective than another, based on all the information in the model, and serves also as the basis for expected value of information (EVI) analyses.

As mentioned in the introduction, the “simple formula” is sometimes used instead to convert from transition rates to probabilities: $p_{\mathrm{ij}}\left(t\right)=1-e^{-q_{\mathrm{ij}}t}$ for i≠j, and $p_{\mathrm{ii}}\left(t\right)=1-\sum_{j\neq i}p_{\mathrm{ij}}\left(t\right)$ so that the rows sum to 1.^25^ This ignores all the transitions except the one from i to j, so it is correct when i is a death state or there is only one transition from state i and that is to a death state, but otherwise it is incorrect. For example, in Model 1 the simple formula is correct for i=2 and j=3 but incorrect for the other two transitions. If $q_{12}=q_{13}=q_{23}=1$ and t is also 1, then the simple formula gives $p_{12}=p_{13}=0.632$ and $p_{11}=1-p_{12}-p_{13}=-0.264$, which is out of bounds for a probability. The correct values are $p_{11}=0.135$, $p_{12}=0.233$, and $p_{23}=0.632$. Sendi and Clemen^26^ proposed a method to avoid probabilities outside the [0, 1] bounds by decomposing a three-way chance node into a sequence of two conditional two-way nodes. This is equivalent to a special case of Model 1 in which $q_{23}=0$, so that states 2 and 3 are competing risks. In this case, $p_{12}=\left(1-e^{q_{11}t}\right)q_{12}/\left(q_{12}+q_{13}\right)$ and $p_{13}=\left(1-e^{q_{11}t}\right)q_{13}/\left(q_{12}+q_{13}\right)$, and in the above example, these formulas give $p_{12}=p_{13}=0.432$ and $p_{11}=0.135$. But they are not valid if $q_{23}>0$.

The question arises of when the simple formula might be approximately correct and sufficient for practical purposes. This happens when all the rates of transitions from i and j are small over the period of one cycle, which is the case if the cycles are sufficiently short. It also happens when there is a state j such that $q_{\mathrm{ij}}$ is much bigger than $q_{\mathrm{ik}}$ for all k≠j, and either j is a death state or both t and all the $q_{\mathrm{jl}}$’s are small—in other words, from state i there is only one transition or “almost” only one transition, and that transition is to a death state or an “almost-death” state. (The simple formula is then approximately correct because the transitions other than from i to j are unlikely to happen.) In any case our procedure should make it easier to use the correct formulas in a wide variety of models.

## Supplementary Material

Supplementary material

Supplementary material

Supplementary material

Supplementary material

Supplementary material

Supplementary material

Supplementary material

Supplementary material

Supplementary material

## References

[1] Briggs AH Ades AE Price MJ Probabilistic sensitivity analysis for decision trees with multiple branches: use of the Dirichlet distribution in a Bayesian framework. Med Decis Making. 2003; 23:341–50. 12926584 10.1177/0272989X03255922

[2] Putter H Fiocco M Geskus RB Tutorial in biostatistics: competing risks and multi-state models. Stat Med. 2007; 26:2389–430. 17031868 10.1002/sim.2712

[3] Welton NJ Ades AE Estimation of Markov chain transition probabilities and rates from fully and partially observed data: uncertainty propagation, evidence synthesis, and model calibration. Med Decis Making. 2005; 25:633–45. 16282214 10.1177/0272989X05282637

[4] Welton NJ Solution to Kolmogorov’s equations for some common Markov models. 2007. Available from: URL: http://www.bristol.ac.uk/social-community-medicine/media/mpes/supplement.pdf. Accessed on 3rd October 2016.

[5] Jackson C Multi-state modelling with R: the msm package. 2016 Available from: URL: https://cran.r-project.org/web/packages/msm/vignettes/msm-manual.pdf. Accessed on 3rd October 2016.

[6] Cox DR Miller HD The Theory of Stochastic Processes. Boca Raton: Chapman and Hall / CRC; 1965.

[7] Grimmett G Stirzaker D Probability and Random Processes. 3rd ed. Oxford: Oxford University Press; 2001.

[8] Chung KL Markov Chains with Stationary Transition Probabilities. 2nd ed. Berlin: Springer-Verlag; 1967.

[9] Freedman D Markov Chains. San Francisco: Holden-Day; 1971.

[10] Turnbull HW Theory of Equations. 3rd ed. Edinburgh: Oliver and Boyd; 1946.

[11] Hellman MJ A unifying technique for the solution of the quadratic, cubic, and quartic. Am Math Mon. 1958; 65:274–6.

[12] Neumark S Solution of Cubic and Quartic Equations. Oxford: Pergamon Press; 1965.

[13] Abel NH Mémoire sur les equations algébriques, où l’on démontre l’impossibilité de la résolution de l’équation générale du cinquième degré. In: Sylow L Lie S, ed. Oeuvres complètes de Niels Henrik Abel. Cambridge: Cambridge University Press; 2012.

[14] Moler C Van Loan C Nineteen dubious ways to compute the exponential of a matrix. SIAM Rev. 1978; 20:801–36.

[15] Moler C Van Loan C Nineteen dubious ways to compute the exponential of a matrix, twenty-five years later. SIAM Rev. 2003; 45:3–49.

[16] Higham NJ Functions of matrices: theory and computation. Philadelphia: Society for Industrial and Applied Mathematics; 2008.

[17] Higham NJ The scaling and squaring method for the matrix exponential revisited. SIAM Rev. 2009; 51:747–64.

[18] Goulet V Dutang C Maechler M et al expm: matrix exponential, R package version 0.999-0. 2015 Available from: URL: https://cran.r-project.org/web/packages/expm/.

[19] Jackson C Multi-state models for panel data: the msm package for R. J Stat Softw. 2011; 38:1–29.

[20] NAG Fortran Library. Mark 25. 2016 Available from: URL: https://www.nag.co.uk/nag-fortran-library/.

[21] Goldberg D What every computer scientist should know about floating-point arithmetic. ACM Comp Surv. 1991; 23:5–48.

[22] IEEE. IEEE standard for floating-point arithmetic. 2008:1–70.

[23] Chiang CL Introduction to Stochastic Processes in Biostatistics. New York: Wiley; 1968.

[24] Maxima, a Computer Algebra System. Version 5.37.3. 2016 Available from: URL: http://maxima.sourceforge.net/.

[25] Fleurence RL Hollenbeak CS Rates and probabilities in economic modelling: transformation, translation and appropriate application. Pharmacoeconomics. 2007; 25:3–6. 17192114 10.2165/00019053-200725010-00002

[26] Sendi PP Clemen RT Sensitivity analysis on a chance node with more than two branches. Med Decis Making. 1999; 19:499–502. 10520688 10.1177/0272989X9901900418

