# Supplementary material for: A Procedure for Deriving Formulas to Convert Transition Rates to Probabilities for Multistate Markov Models
Source: Med Decis Making. 2017 Apr 5;37(7):779–89. doi: 10.1177/0272989X17696997 (PMC5582645; doi:10.1177/0272989X17696997)
Supplement: Supplementary material [file Model_1,_redesigned_for_PDF.rjf_online_supp.pdf]

Supplementary file for Jones, Epstein, Garcia-Mochon, "A procedure for deriving formulas to convert transition rates to probabilities for multi-state Markov models"

Model 1. Three-state model with forward transitions only

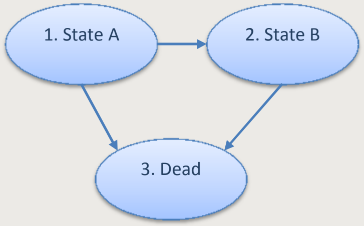

Matrix Q

	State A	State B	Dead
State A	-0.01	0.002996	0.0023346
State B	0.00	0.00	0.00099
Dead	0.00	0.00	0.00

Matrix D

	State A	State B	Dead
State A	-0.0053	0	0
State B	0	-0.0010	0
Dead	0	0	0

Matrix U

	State A	State B	Dead
State A	1.0000	0.00	1
State B	0	0.0043	1
Dead	0	0	1

Matrix U^(-1)

	State A	State B	Dead
State A	1.00	-0.69	-0.31
State B	0.00	230.57	-230.57
Dead	0.00	0.00	1.00

Matrix Exp(D)

	State A	State B	Dead
State A	0.9947	0	0
State B	0	0.9990	0
Dead	0	0	1

Matrix P(1) = U * Exp(D) * U^(-1)

	State A	State B	Dead
State A	0.995	0.003	0.002
State B	0.000	0.999	0.001
Dead	0.000	0.000	1.000

	Transition rates					*Transition probabilities*					State transition model (Markov trace)			
	From State A to ...			From State B to ...		From State A to ...			From State B to ...					
	State A	State B	Dead	State B	Dead	State A	State B	Dead	State B	Dead	State A	State B	Dead	*Check*
Cycle	q11	q12	q13	-q23	q23									
0											1			
1	-0.01	0.0029957	0.0023346	-0.000993	0.00099	0.995	0.003	0.002	0.999	0.001	0.995	0.003	0.002	1.00
2	-0.01	0.0036877	0.0027034	-0.001785	0.00179	0.994	0.004	0.003	0.998	0.002	0.988	0.007	0.005	1.00
3	-0.01	0.0043751	0.0030449	-0.002386	0.00239	0.993	0.004	0.003	0.998	0.002	0.981	0.011	0.008	1.00
4	-0.01	0.0050513	0.0033702	-0.003002	0.00300	0.992	0.005	0.003	0.997	0.003	0.973	0.016	0.011	1.00
5	-0.01	0.0057231	0.0036938	-0.003714	0.00371	0.991	0.006	0.004	0.996	0.004	0.964	0.021	0.015	1.00
6	-0.01	0.0064007	0.0040325	-0.004503	0.00450	0.990	0.006	0.004	0.996	0.004	0.954	0.028	0.019	1.00
7	-0.01	0.0070966	0.0044027	-0.005341	0.00534	0.989	0.007	0.004	0.995	0.005	0.943	0.034	0.023	1.00
8	-0.01	0.0078172	0.0048117	-0.006204	0.00620	0.987	0.008	0.005	0.994	0.006	0.931	0.041	0.028	1.00
9	-0.01	0.0085558	0.0052566	-0.007073	0.00707	0.986	0.009	0.005	0.993	0.007	0.918	0.049	0.033	1.00
10	-0.02	0.009298	0.0057349	-0.007943	0.00794	0.985	0.009	0.006	0.992	0.008	0.904	0.057	0.038	1.00
11	-0.02	0.0100377	0.0062397	-0.008797	0.00880	0.984	0.010	0.006	0.991	0.009	0.890	0.066	0.044	1.00
12	-0.02	0.0107798	0.0067639	-0.009632	0.00963	0.983	0.011	0.007	0.990	0.010	0.874	0.075	0.051	1.00
13	-0.02	0.0115252	0.007309	-0.010455	0.01045	0.981	0.012	0.007	0.990	0.010	0.858	0.084	0.058	1.00
14	-0.02	0.0122756	0.0078889	-0.011283	0.01128	0.980	0.012	0.008	0.989	0.011	0.841	0.094	0.065	1.00
15	-0.02	0.0130302	0.0085212	-0.012136	0.01214	0.979	0.013	0.008	0.988	0.012	0.823	0.104	0.073	1.00
16	-0.02	0.0137858	0.0092185	-0.013032	0.01303	0.977	0.014	0.009	0.987	0.013	0.804	0.114	0.082	1.00
17	-0.02	0.0145385	0.0099915	-0.014013	0.01401	0.976	0.015	0.010	0.986	0.014	0.785	0.124	0.091	1.00
18	-0.03	0.0152881	0.0108583	-0.015122	0.01512	0.974	0.015	0.010	0.985	0.015	0.765	0.134	0.101	1.00
19	-0.03	0.0160405	0.0118465	-0.016375	0.01638	0.972	0.016	0.011	0.984	0.016	0.744	0.144	0.112	1.00
20	-0.03	0.0167826	0.0129892	-0.01778	0.01778	0.971	0.017	0.012	0.982	0.018	0.722	0.154	0.124	1.00
21	-0.03	0.0174968	0.0142995	-0.019324	0.01932	0.969	0.018	0.014	0.981	0.019	0.699	0.164	0.137	1.00
22	-0.03	0.0181637	0.0157778	-0.020995	0.02099	0.967	0.018	0.015	0.979	0.021	0.676	0.173	0.151	1.00
23	-0.04	0.0187573	0.0174253	-0.022768	0.02277	0.964	0.019	0.017	0.977	0.023	0.652	0.182	0.166	1.00
24	-0.04	0.0192367	0.0192662	-0.024639	0.02464	0.962	0.019	0.018	0.976	0.024	0.627	0.191	0.182	1.00
25	-0.04	0.0195705	0.0213433	-0.026604	0.02660	0.960	0.020	0.020	0.974	0.026	0.602	0.198	0.200	1.00
26	-0.04	0.0197615	0.0236544	-0.028664	0.02866	0.958	0.020	0.023	0.972	0.028	0.576	0.204	0.219	1.00
27	-0.05	0.0198191	0.0261833	-0.030823	0.03082	0.955	0.020	0.025	0.970	0.030	0.551	0.210	0.240	1.00
28	-0.05	0.019745	0.0289248	-0.033096	0.03310	0.952	0.020	0.028	0.967	0.033	0.524	0.214	0.262	1.00
29	-0.05	0.0195386	0.0318917	-0.035485	0.03548	0.950	0.020	0.030	0.965	0.035	0.498	0.217	0.285	1.00
30	-0.05	0.0191978	0.0351053	-0.037976	0.03798	0.947	0.019	0.033	0.963	0.037	0.472	0.218	0.310	1.00
31	-0.06	0.0187206	0.0385895	-0.040544	0.04054	0.944	0.019	0.037	0.960	0.040	0.445	0.218	0.336	1.00
32	-0.06	0.0180982	0.0423698	-0.043163	0.04316	0.941	0.018	0.040	0.958	0.042	0.419	0.217	0.363	1.00
33	-0.06	0.0173071	0.0464756	-0.045829	0.04583	0.938	0.017	0.044	0.955	0.045	0.393	0.215	0.392	1.00
34	-0.07	0.0163075	0.0509314	-0.048526	0.04853	0.935	0.016	0.049	0.953	0.047	0.368	0.211	0.421	1.00
35	-0.07	0.0150387	0.0557517	-0.051202	0.05120	0.932	0.015	0.053	0.950	0.050	0.343	0.206	0.451	1.00
36	-0.07	0.0134288	0.0609449	-0.0538	0.05380	0.928	0.014	0.058	0.948	0.052	0.318	0.200	0.482	1.00
37	-0.08	0.0114066	0.0665358	-0.056247	0.05625	0.925	0.012	0.063	0.945	0.055	0.294	0.193	0.513	1.00
38	-0.08	0.008918	0.0725969	-0.058481	0.05848	0.922	0.009	0.069	0.943	0.057	0.271	0.185	0.544	1.00
