# Supplementary material for: A Procedure for Deriving Formulas to Convert Transition Rates to Probabilities for Multistate Markov Models
Source: Med Decis Making. 2017 Apr 5;37(7):779–89. doi: 10.1177/0272989X17696997 (PMC5582645; doi:10.1177/0272989X17696997)
Supplement: Supplementary material [file Model_2,_redesigned_for_PDF.rjf_online_supp.pdf]

Supplementary file to accompany Jones, Epstein, Garcia-Mochon "A procedure for deriving formulas to convert transition rates to probabilities for multi-state Markov models"

Model 2. Four-state model with forward transitions only

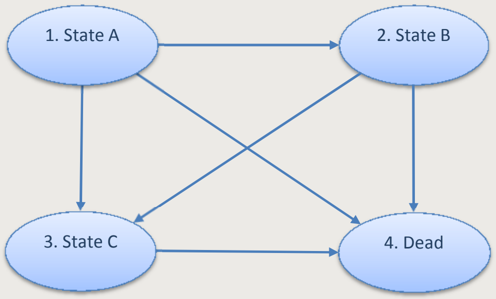

Matrix Q

	State A	State B	State C	Dead
State A	-0.006	0.0030	0.0006	0.0023
State B	0	-0.054	0.0532	0.0010
State C	0	0	-0.02	0.02
Dead	0	0	0	0

Matrix D

	State A	State B	State C	Dead
State A	-0.006	0	0	0
State B	0	-0.0542	0	0
State C	0	0	-0.0200	0
Dead	0	0	0	0

Matrix U

	State A	State B	State C	Dead
State A	1	0.0029957	0.000	0.000
State B	0	-0.0483	-0.0003	0.000
State C	0	0	-0.0003	0.000
Dead	0	0	0	0.000

Matrix U^(-1)

	State A	State B	State C	Dead
State A	1	0.062	-0.792	-0.270
State B	0	-20.723	20.726	-0.002
State C	0	0	-3799.755	3799.755
Dead	0	0	0	3102.472

	Transition rates									*Transition probabilities*					
	From State A to ...				From State B to ...			From State C to Dead		From State A to ...				From State B to ...	
	State A	State B	State B	Dead	State B	State C	Dead	State C	Dead	State A	State B	State C	Dead	State B	State C
Cycle	a	b	c	d	f	g	h	-i	i						
0															
1	-0.0059	0.0030	0.0006	0.0023	-0.054	0.0532	0.0010	-0.001	0.001	0.9941	0.0029	0.0007	0.0023	0.9472	0.0518
2	-0.0072	0.0037	0.0008	0.0027	-0.059	0.0574	0.0018	-0.001	0.001	0.9929	0.0036	0.0009	0.0027	0.9425	0.0557
3	-0.0084	0.0044	0.0009	0.0030	-0.063	0.0603	0.0024	-0.001	0.001	0.9917	0.0042	0.0011	0.0030	0.9392	0.0584
4	-0.0095	0.0051	0.0011	0.0034	-0.065	0.0624	0.0031	-0.001	0.001	0.9905	0.0049	0.0013	0.0034	0.9367	0.0603
5	-0.0107	0.0057	0.0013	0.0037	-0.068	0.0640	0.0038	-0.001	0.001	0.9894	0.0055	0.0015	0.0037	0.9345	0.0618
6	-0.0119	0.0064	0.0015	0.0040	-0.070	0.0654	0.0046	-0.001	0.001	0.9882	0.0061	0.0017	0.0040	0.9323	0.0632
7	-0.0132	0.0071	0.0017	0.0044	-0.072	0.0668	0.0055	-0.001	0.001	0.9869	0.0068	0.0019	0.0044	0.9302	0.0644
8	-0.0145	0.0078	0.0019	0.0048	-0.075	0.0683	0.0064	-0.001	0.001	0.9856	0.0075	0.0021	0.0048	0.9280	0.0658
9	-0.0159	0.0086	0.0021	0.0053	-0.077	0.0696	0.0073	-0.001	0.001	0.9842	0.0082	0.0024	0.0052	0.9259	0.0670
10	-0.0174	0.0093	0.0023	0.0057	-0.079	0.0708	0.0082	-0.001	0.001	0.9828	0.0089	0.0026	0.0057	0.9241	0.0680
11	-0.0189	0.0100	0.0026	0.0062	-0.081	0.0715	0.0091	-0.001	0.001	0.9813	0.0096	0.0029	0.0062	0.9226	0.0686
12	-0.0204	0.0108	0.0029	0.0068	-0.082	0.0717	0.0100	-0.001	0.001	0.9798	0.0102	0.0032	0.0067	0.9216	0.0688
13	-0.0220	0.0115	0.0032	0.0073	-0.082	0.0713	0.0109	-0.001	0.001	0.9783	0.0109	0.0035	0.0073	0.9211	0.0684
14	-0.0236	0.0123	0.0035	0.0079	-0.082	0.0705	0.0117	-0.001	0.001	0.9766	0.0116	0.0039	0.0079	0.9211	0.0677
15	-0.0254	0.0130	0.0038	0.0085	-0.082	0.0693	0.0126	-0.001	0.001	0.9749	0.0124	0.0042	0.0085	0.9213	0.0665
16	-0.0272	0.0138	0.0042	0.0092	-0.081	0.0680	0.0135	-0.001	0.001	0.9732	0.0131	0.0046	0.0092	0.9217	0.0652
17	-0.0291	0.0145	0.0046	0.0100	-0.081	0.0665	0.0146	-0.001	0.001	0.9713	0.0138	0.0050	0.0100	0.9222	0.0638
18	-0.0312	0.0153	0.0050	0.0109	-0.081	0.0651	0.0157	-0.001	0.001	0.9693	0.0145	0.0054	0.0108	0.9224	0.0625
19	-0.0333	0.0160	0.0054	0.0118	-0.081	0.0638	0.0170	-0.001	0.001	0.9672	0.0152	0.0058	0.0118	0.9224	0.0613
20	-0.0357	0.0168	0.0059	0.0130	-0.081	0.0628	0.0185	-0.001	0.001	0.9649	0.0158	0.0063	0.0129	0.9220	0.0602
21	-0.0382	0.0175	0.0064	0.0143	-0.082	0.0620	0.0201	-0.001	0.001	0.9625	0.0165	0.0068	0.0142	0.9211	0.0595
22	-0.0410	0.0182	0.0070	0.0158	-0.084	0.0618	0.0219	-0.001	0.001	0.9598	0.0171	0.0074	0.0157	0.9198	0.0592
23	-0.0439	0.0188	0.0077	0.0174	-0.086	0.0619	0.0237	-0.001	0.001	0.9570	0.0176	0.0081	0.0173	0.9180	0.0593
24	-0.0470	0.0192	0.0085	0.0193	-0.088	0.0623	0.0257	-0.001	0.001	0.9541	0.0180	0.0089	0.0191	0.9158	0.0596
25	-0.0504	0.0196	0.0094	0.0213	-0.091	0.0631	0.0278	-0.001	0.001	0.9509	0.0182	0.0098	0.0211	0.9131	0.0603
26	-0.0540	0.0198	0.0106	0.0237	-0.095	0.0648	0.0300	-0.001	0.001	0.9475	0.0183	0.0109	0.0233	0.9096	0.0618
27	-0.0578	0.0198	0.0118	0.0262	-0.100	0.0673	0.0324	-0.001	0.001	0.9438	0.0183	0.0121	0.0258	0.9051	0.0640
28	-0.0619	0.0197	0.0133	0.0289	-0.106	0.0709	0.0348	-0.001	0.001	0.9399	0.0182	0.0135	0.0284	0.8996	0.0673
29	-0.0663	0.0195	0.0149	0.0319	-0.113	0.0757	0.0375	-0.001	0.001	0.9359	0.0179	0.0151	0.0312	0.8929	0.0716
30	-0.0709	0.0192	0.0166	0.0351	-0.122	0.0818	0.0403	-0.001	0.001	0.9315	0.0174	0.0168	0.0343	0.8850	0.0770
31	-0.0759	0.0187	0.0185	0.0386	-0.133	0.0893	0.0432	-0.001	0.001	0.9270	0.0169	0.0186	0.0375	0.8758	0.0836
32	-0.0811	0.0181	0.0207	0.0424	-0.145	0.0984	0.0463	-0.001	0.001	0.9221	0.0162	0.0207	0.0411	0.8653	0.0916
33	-0.0868	0.0173	0.0230	0.0465	-0.159	0.1091	0.0495	-0.001	0.001	0.9169	0.0153	0.0229	0.0449	0.8533	0.1009
34	-0.0929	0.0163	0.0256	0.0509	-0.174	0.1215	0.0528	-0.001	0.001	0.9113	0.0143	0.0254	0.0490	0.8401	0.1114
35	-0.0994	0.0150	0.0286	0.0558	-0.192	0.1355	0.0562	-0.001	0.001	0.9054	0.0130	0.0281	0.0535	0.8256	0.1232
36	-0.1063	0.0134	0.0319	0.0609	-0.211	0.1514	0.0596	-0.001	0.001	0.8991	0.0115	0.0312	0.0582	0.8098	0.1364
37	-0.1137	0.0114	0.0358	0.0665	-0.232	0.1695	0.0629	-0.001	0.001	0.8925	0.0096	0.0347	0.0632	0.7926	0.1511
38	-0.1218	0.0089	0.0403	0.0726	-0.257	0.1904	0.0662	-0.001	0.001	0.8853	0.0074	0.0386	0.0686	0.7737	0.1679

Matrix Exp(D)

	State A	State B	State C	Dead
State A	0.994070947	0	0	0
State B	0	0.947241	0	0
State C	0	0	0.980199	0
Dead	0	0	0	1.000000

Matrix P(1) = U * Exp(D) * U^(-1)

	State A	State B	State C	Dead	
State A	0.9941	0.0029	-0.0130	0.0161	1.00000
State B	0	0.947241	0.032962	0.019797	1.00000
State C	0	0	0.980199	0.019801	1.00000
Dead	0	0	0	1.000000	1.00000

Intermediate variables

	From State C to …		State transition model (Markov trace)					U=					
**Dead**	**State C**	**Dead**	**State A**	**State B**	**State C**	**Dead**	*Check*	1	b	j	k	l	m
			1	0	0	0	1.00						
0.0010	0.9990	0.0010	0.994	0.003	0.001	0.002	1.00		0.00300	-0.00019	0.00032	-0.04826	-0.00026
0.0018	0.9990	0.0010	0.987	0.006	0.002	0.005	1.00		0.00369	-0.00026	0.00042	-0.05207	-0.00035
0.0024	0.9990	0.0010	0.979	0.010	0.003	0.008	1.00		0.00438	-0.00032	0.00052	-0.05438	-0.00044
0.0030	0.9990	0.0010	0.969	0.014	0.005	0.011	1.00		0.00505	-0.00039	0.00062	-0.05590	-0.00053
0.0037	0.9990	0.0010	0.959	0.019	0.007	0.015	1.00		0.00572	-0.00045	0.00073	-0.05709	-0.00062
0.0045	0.9990	0.0010	0.948	0.023	0.010	0.019	1.00		0.00640	-0.00052	0.00083	-0.05816	-0.00071
0.0053	0.9990	0.0010	0.935	0.028	0.013	0.023	1.00		0.00710	-0.00059	0.00095	-0.05918	-0.00081
0.0062	0.9990	0.0010	0.922	0.033	0.017	0.028	1.00		0.00782	-0.00067	0.00108	-0.06018	-0.00092
0.0071	0.9990	0.0010	0.907	0.038	0.021	0.033	1.00		0.00856	-0.00075	0.00122	-0.06105	-0.00104
0.0079	0.9990	0.0010	0.892	0.043	0.026	0.039	1.00		0.00930	-0.00084	0.00137	-0.06163	-0.00116
0.0088	0.9990	0.0010	0.875	0.048	0.032	0.044	1.00		0.01004	-0.00092	0.00152	-0.06173	-0.00128
0.0096	0.9990	0.0010	0.857	0.054	0.038	0.051	1.00		0.01078	-0.00100	0.00167	-0.06127	-0.00139
0.0105	0.9990	0.0010	0.839	0.059	0.045	0.058	1.00		0.01153	-0.00108	0.00181	-0.06020	-0.00150
0.0113	0.9990	0.0010	0.819	0.064	0.052	0.065	1.00		0.01228	-0.00115	0.00194	-0.05859	-0.00160
0.0121	0.9990	0.0010	0.799	0.069	0.060	0.073	1.00		0.01303	-0.00121	0.00208	-0.05656	-0.00169
0.0130	0.9990	0.0010	0.777	0.074	0.068	0.081	1.00		0.01379	-0.00128	0.00222	-0.05428	-0.00178
0.0140	0.9990	0.0010	0.755	0.079	0.076	0.090	1.00		0.01454	-0.00133	0.00236	-0.05191	-0.00187
0.0151	0.9990	0.0010	0.732	0.084	0.085	0.099	1.00		0.01529	-0.00139	0.00252	-0.04962	-0.00196
0.0164	0.9990	0.0010	0.708	0.088	0.094	0.109	1.00		0.01604	-0.00146	0.00269	-0.04749	-0.00206
0.0178	0.9990	0.0010	0.683	0.093	0.104	0.120	1.00		0.01678	-0.00153	0.00290	-0.04554	-0.00218
0.0193	0.9990	0.0010	0.657	0.097	0.114	0.132	1.00		0.01750	-0.00161	0.00314	-0.04390	-0.00231
0.0210	0.9990	0.0010	0.631	0.100	0.125	0.144	1.00		0.01816	-0.00170	0.00343	-0.04263	-0.00247
0.0228	0.9990	0.0010	0.604	0.103	0.136	0.158	1.00		0.01876	-0.00181	0.00376	-0.04169	-0.00265
0.0246	0.9990	0.0010	0.576	0.105	0.147	0.172	1.00		0.01924	-0.00194	0.00414	-0.04099	-0.00287
0.0266	0.9990	0.0010	0.548	0.106	0.159	0.187	1.00		0.01957	-0.00208	0.00458	-0.04059	-0.00312
0.0287	0.9990	0.0010	0.519	0.107	0.171	0.203	1.00		0.01976	-0.00227	0.00511	-0.04080	-0.00343
0.0308	0.9990	0.0010	0.490	0.106	0.184	0.220	1.00		0.01982	-0.00250	0.00576	-0.04184	-0.00383
0.0331	0.9990	0.0010	0.460	0.105	0.198	0.237	1.00		0.01975	-0.00279	0.00655	-0.04385	-0.00432
0.0355	0.9990	0.0010	0.431	0.102	0.212	0.256	1.00		0.01954	-0.00315	0.00751	-0.04694	-0.00495
0.0380	0.9990	0.0010	0.401	0.097	0.227	0.274	1.00		0.01920	-0.00358	0.00866	-0.05120	-0.00572
0.0405	0.9990	0.0010	0.372	0.092	0.242	0.294	1.00		0.01872	-0.00411	0.01006	-0.05674	-0.00669
0.0432	0.9990	0.0010	0.343	0.086	0.258	0.313	1.00		0.01810	-0.00475	0.01174	-0.06360	-0.00789
0.0458	0.9990	0.0010	0.315	0.078	0.274	0.333	1.00		0.01731	-0.00552	0.01377	-0.07184	-0.00936
0.0485	0.9990	0.0010	0.287	0.070	0.291	0.352	1.00		0.01631	-0.00642	0.01619	-0.08142	-0.01116
0.0512	0.9990	0.0010	0.260	0.062	0.307	0.371	1.00		0.01504	-0.00749	0.01905	-0.09232	-0.01333
0.0538	0.9990	0.0010	0.233	0.053	0.323	0.390	1.00		0.01343	-0.00874	0.02243	-0.10464	-0.01594
0.0562	0.9990	0.0010	0.208	0.044	0.339	0.408	1.00		0.01141	-0.01022	0.02643	-0.11866	-0.01910
0.0585	0.9990	0.0010	0.184	0.036	0.354	0.425	1.00		0.00892	-0.01199	0.03125	-0.13485	-0.02300

			Uinverse												
n	o	p		q	r	s	u	v	w	x	y	z	expa	expff	exp-i
0.00032	-0.00026	0.00032	1	0.06208	-0.79236	-0.26972	-20.72307	20.72556	-0.00249	-3799.75550	3799.75550	3102.47190	0.99407095	0.94724065	0.9990005
0.00042	-0.00036	0.00042	1	0.07082	-0.78543	-0.28539	-19.20503	18.93809	0.26694	-2783.13729	2783.13729	2354.51763	0.99285614	0.9424813	0.9990005
0.00052	-0.00045	0.00052	1	0.08045	-0.78708	-0.29337	-18.38773	17.96153	0.42620	-2199.59051	2199.59051	1905.70650	0.99167207	0.93918115	0.9990005
0.00062	-0.00055	0.00062	1	0.09036	-0.79067	-0.29969	-17.88864	17.31412	0.57452	-1818.80538	1818.80538	1603.13281	0.99051239	0.93666058	0.9990005
0.00073	-0.00065	0.00073	1	0.10025	-0.79357	-0.30667	-17.51617	16.77965	0.73653	-1543.16950	1543.16950	1378.33875	0.98935506	0.93445473	0.9990005
0.00083	-0.00075	0.00083	1	0.11005	-0.79521	-0.31484	-17.19413	16.29043	0.90370	-1328.38212	1328.38212	1199.39162	0.98816993	0.93233788	0.9990005
0.00095	-0.00087	0.00095	1	0.11991	-0.79568	-0.32422	-16.89632	15.82986	1.06645	-1152.63920	1152.63920	1050.33271	0.98692592	0.9302101	0.9990005
0.00108	-0.00099	0.00108	1	0.12990	-0.79544	-0.33446	-16.61679	15.39808	1.21871	-1005.77020	1005.77020	923.84436	0.98560935	0.92804477	0.9990005
0.00122	-0.00113	0.00122	1	0.14014	-0.79502	-0.34512	-16.37977	15.01820	1.36157	-883.84974	883.84974	817.48814	0.98422849	0.92593791	0.9990005
0.00137	-0.00128	0.00137	1	0.15088	-0.79466	-0.35622	-16.22713	14.72364	1.50349	-784.26256	784.26256	729.70736	0.98279768	0.92406099	0.9990005
0.00152	-0.00142	0.00152	1	0.16260	-0.79511	-0.36748	-16.19861	14.54595	1.65266	-703.89981	703.89981	658.28787	0.98132567	0.92257685	0.9990005
0.00167	-0.00156	0.00167	1	0.17595	-0.79719	-0.37876	-16.32211	14.50205	1.82006	-639.16068	639.16068	600.38035	0.9798104	0.92158266	0.9990005
0.00181	-0.00170	0.00181	1	0.19146	-0.80100	-0.39046	-16.61234	14.59599	2.01635	-586.82411	586.82411	553.32255	0.97825012	0.92110053	0.9990005
0.00194	-0.00184	0.00194	1	0.20953	-0.80619	-0.40334	-17.06872	14.81678	2.25194	-543.56086	543.56086	514.24418	0.9766302	0.9210565	0.9990005
0.00208	-0.00197	0.00208	1	0.23039	-0.81232	-0.41808	-17.68159	15.14645	2.53514	-506.63721	506.63721	480.74012	0.974934	0.92132585	0.9990005
0.00222	-0.00211	0.00222	1	0.25397	-0.81896	-0.43501	-18.42275	15.55371	2.86904	-473.98098	473.98098	450.95983	0.97315569	0.92174018	0.9990005
0.00236	-0.00225	0.00236	1	0.28005	-0.82541	-0.45464	-19.26292	16.00070	3.26223	-444.19177	444.19177	423.64859	0.9712932	0.92215671	0.9990005
0.00252	-0.00241	0.00252	1	0.30812	-0.83091	-0.47721	-20.15422	16.43831	3.71591	-415.72094	415.72094	397.39535	0.96932599	0.92240425	0.9990005
0.00269	-0.00258	0.00269	1	0.33778	-0.83502	-0.50276	-21.05782	16.83325	4.22456	-387.47080	387.47080	371.19614	0.96721662	0.92235869	0.9990005
0.00290	-0.00278	0.00290	1	0.36850	-0.83726	-0.53124	-21.95722	17.17375	4.78347	-359.23412	359.23412	344.87155	0.9649359	0.92197543	0.9990005
0.00314	-0.00302	0.00314	1	0.39859	-0.83714	-0.56145	-22.78076	17.41925	5.36151	-330.89326	330.89326	318.31833	0.96247711	0.92114142	0.9990005
0.00343	-0.00330	0.00343	1	0.42604	-0.83425	-0.59179	-23.45535	17.53503	5.92032	-302.71785	302.71785	291.79970	0.95984449	0.91978236	0.9990005
0.00376	-0.00363	0.00376	1	0.44996	-0.82873	-0.62123	-23.98852	17.54378	6.44474	-275.57874	275.57874	266.15473	0.95704994	0.91797392	0.9990005
0.00414	-0.00400	0.00414	1	0.46927	-0.82013	-0.64914	-24.39441	17.46606	6.92835	-249.89600	249.89600	241.79952	0.95408749	0.91576738	0.9990005
0.00458	-0.00444	0.00458	1	0.48212	-0.80808	-0.67404	-24.63491	17.29451	7.34040	-225.28811	225.28811	218.38568	0.95089598	0.91306939	0.9990005
0.00511	-0.00497	0.00511	1	0.48431	-0.79135	-0.69296	-24.50758	16.92557	7.58201	-201.33523	201.33523	195.51949	0.9474628	0.90958093	0.9990005
0.00576	-0.00561	0.00576	1	0.47368	-0.76931	-0.70437	-23.89995	16.30678	7.59317	-178.30607	178.30607	173.46517	0.94380519	0.90513009	0.9990005
0.00655	-0.00639	0.00655	1	0.45033	-0.74203	-0.70831	-22.80746	15.44170	7.36576	-156.59993	156.59993	152.61525	0.93993881	0.89961733	0.9990005
0.00751	-0.00733	0.00751	1	0.41626	-0.71048	-0.70578	-21.30456	14.37716	6.92741	-136.46440	136.46440	133.21890	0.93585662	0.8929441	0.9990005
0.00866	-0.00847	0.00866	1	0.37493	-0.67640	-0.69854	-19.52999	13.19308	6.33691	-118.08384	118.08384	115.46546	0.93153828	0.88504101	0.9990005
0.01006	-0.00985	0.01006	1	0.32996	-0.64153	-0.68842	-17.62537	11.96677	5.65860	-101.52959	101.52959	99.43538	0.92695478	0.87582682	0.9990005
0.01174	-0.01152	0.01174	1	0.28455	-0.60735	-0.67719	-15.72243	10.76597	4.95646	-86.82699	86.82699	85.16424	0.92207464	0.86525372	0.9990005
0.01377	-0.01352	0.01377	1	0.24093	-0.57466	-0.66627	-13.92069	9.63714	4.28355	-73.94552	73.94552	72.63276	0.91686668	0.85331314	0.9990005
0.01619	-0.01592	0.01619	1	0.20030	-0.54384	-0.65646	-12.28267	8.61002	3.67265	-62.81416	62.81416	61.78127	0.91131114	0.84005627	0.9990005
0.01905	-0.01876	0.01905	1	0.16290	-0.51492	-0.64797	-10.83181	7.69653	3.13528	-53.30743	53.30743	52.49570	0.90540493	0.8255597	0.9990005
0.02243	-0.02211	0.02243	1	0.12833	-0.48771	-0.64063	-9.55669	6.88947	2.66723	-45.22844	45.22844	44.59062	0.89914506	0.80981488	0.9990005
0.02643	-0.02609	0.02643	1	0.09613	-0.46198	-0.63415	-8.42720	6.17115	2.25605	-38.33397	38.33397	37.83342	0.89249488	0.79263067	0.9990005
0.03125	-0.03087	0.03125	1	0.06613	-0.43762	-0.62851	-7.41541	5.52388	1.89153	-32.38959	32.38959	31.99844	0.8853464	0.77365395	0.9990005
