# Supplementary material for: A Procedure for Deriving Formulas to Convert Transition Rates to Probabilities for Multistate Markov Models
Source: Med Decis Making. 2017 Apr 5;37(7):779–89. doi: 10.1177/0272989X17696997 (PMC5582645; doi:10.1177/0272989X17696997)
Supplement: Supplementary material [file Model_3,_redesigned_for_PDF.rjf_online_supp.pdf]

Supplementary file to accompany Jones, Epstein, Garcia-Mochon "A procedure for deriving formulas to convert transition rates to probabilities for multi-state Markov models"

Model 3. Four-state model with all forward transition and one backward transition

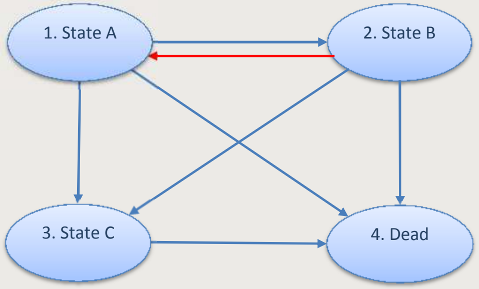

Matrix Q

	State A	State B	State C	Dead
State A	-0.006	0.0030	0.0006	0.0023
State B	0.05321	-0.002	0.00099	0.00100
State C	0	0	0.00	0.00102
Dead	0	0	0	0

Matrix D

	State A	State B	State C	Dead
State A	-0.003	0	0	0
State B	0	-0.0582	0	0
State C	0	0	-0.0010	0
Dead	0	0	0	0

Matrix U

	State A	State B	State C	Dead
State A	0.0060	0.0060	0.0000	-0.0002
State B	0.0061	-0.1046	0.0000	-0.0002
State C	0.0000	0.0000	-0.0001	-0.0002
Dead	0.0000	0.0000	0.0000	-0.0002

Matrix U^(-1)

	State A	State B	State C	Dead
State A	157.7162	9.0333	-56.6521	-110.0974
State B	9.1897	-9.0333	0.0579	-0.2143
State C	0.0000	0.0000	-9322.6497	9322.6497
Dead	0.0000	0.0000	0.0000	-5921.5878

	Transition rates										*Transition probabilities*					
	From State A to ...				From State B to ...				From State C to Dead		From State A to ...				From St	
	State A	State B	State B	Dead	State A	State B	State C	Dead	State C	Dead	State A	State B	State C	Dead	State A	State B
Cycle	a	b	c	d	f	g	h	i	-j	j						
0																
1	-0.00595	0.0030	0.0006	0.0023	0.05321	-0.05520	0.00099	0.00100	0.00	0.00102	0.9941	0.0029	0.0006	0.0023	0.0516	0.9464
2	-0.00717	0.0037	0.0008	0.0027	0.05743	-0.06024	0.00181	0.00100	0.00	0.00278	0.9930	0.0036	0.0008	0.0027	0.0555	0.9416
3	-0.00836	0.0044	0.0009	0.0030	0.06032	-0.06375	0.00243	0.00100	0.00	0.00350	0.9918	0.0042	0.0009	0.0030	0.0582	0.9384
4	-0.00953	0.0051	0.0011	0.0034	0.06236	-0.06643	0.00307	0.00100	0.00	0.00440	0.9907	0.0049	0.0011	0.0034	0.0601	0.9359
5	-0.01070	0.0057	0.0013	0.0037	0.06398	-0.06879	0.00381	0.00100	-0.01	0.00529	0.9895	0.0055	0.0013	0.0037	0.0615	0.9337
6	-0.01190	0.0064	0.0015	0.0040	0.06543	-0.07106	0.00463	0.00100	-0.01	0.00612	0.9884	0.0061	0.0015	0.0040	0.0628	0.9316
7	-0.01316	0.0071	0.0017	0.0044	0.06684	-0.07334	0.00550	0.00100	-0.01	0.00703	0.9872	0.0068	0.0017	0.0044	0.0640	0.9295
8	-0.01450	0.0078	0.0019	0.0048	0.06827	-0.07568	0.00640	0.00100	-0.01	0.00797	0.9859	0.0075	0.0019	0.0048	0.0653	0.9274
9	-0.01590	0.0086	0.0021	0.0053	0.06963	-0.07795	0.00731	0.00100	-0.01	0.00950	0.9845	0.0082	0.0021	0.0052	0.0665	0.9253
10	-0.01735	0.0093	0.0023	0.0057	0.07075	-0.07998	0.00822	0.00100	-0.01	0.01123	0.9831	0.0089	0.0023	0.0057	0.0674	0.9234
11	-0.01885	0.0100	0.0026	0.0062	0.07147	-0.08158	0.00912	0.00100	-0.01	0.01230	0.9817	0.0095	0.0026	0.0062	0.0680	0.9220
12	-0.02040	0.0108	0.0029	0.0068	0.07167	-0.08266	0.00999	0.00100	-0.01	0.01389	0.9802	0.0102	0.0029	0.0067	0.0681	0.9210
13	-0.02199	0.0115	0.0032	0.0073	0.07133	-0.08319	0.01085	0.00100	-0.01	0.01452	0.9786	0.0109	0.0032	0.0073	0.0677	0.9206
14	-0.02365	0.0123	0.0035	0.0079	0.07052	-0.08323	0.01172	0.00100	-0.01	0.01480	0.9770	0.0116	0.0035	0.0078	0.0669	0.9205
15	-0.02539	0.0130	0.0038	0.0085	0.06934	-0.08294	0.01261	0.00100	-0.02	0.01521	0.9754	0.0123	0.0038	0.0085	0.0657	0.9208
16	-0.02721	0.0138	0.0042	0.0092	0.06796	-0.08249	0.01354	0.00100	-0.02	0.01673	0.9736	0.0131	0.0042	0.0091	0.0643	0.9213
17	-0.02913	0.0145	0.0046	0.0100	0.06649	-0.08204	0.01456	0.00100	-0.02	0.01862	0.9718	0.0138	0.0046	0.0099	0.0629	0.9217
18	-0.03115	0.0153	0.0050	0.0109	0.06506	-0.08177	0.01571	0.00100	-0.02	0.02012	0.9698	0.0145	0.0050	0.0108	0.0615	0.9219
19	-0.03333	0.0160	0.0054	0.0118	0.06381	-0.08182	0.01701	0.00100	-0.02	0.02253	0.9677	0.0151	0.0054	0.0117	0.0603	0.9219
20	-0.03569	0.0168	0.0059	0.0130	0.06276	-0.08224	0.01848	0.00100	-0.03	0.02528	0.9654	0.0158	0.0059	0.0128	0.0592	0.9215
21	-0.03825	0.0175	0.0064	0.0143	0.06204	-0.08314	0.02010	0.00100	-0.03	0.02829	0.9630	0.0165	0.0064	0.0141	0.0584	0.9207
22	-0.04098	0.0182	0.0070	0.0158	0.06176	-0.08462	0.02185	0.00100	-0.03	0.03074	0.9604	0.0171	0.0070	0.0156	0.0580	0.9194
23	-0.04390	0.0188	0.0077	0.0174	0.06186	-0.08659	0.02372	0.00100	-0.03	0.03219	0.9576	0.0176	0.0076	0.0172	0.0580	0.9176
24	-0.04700	0.0192	0.0085	0.0193	0.06229	-0.08899	0.02571	0.00100	-0.03	0.03344	0.9547	0.0180	0.0084	0.0190	0.0582	0.9154
25	-0.05035	0.0196	0.0094	0.0213	0.06314	-0.09194	0.02780	0.00100	-0.03	0.03435	0.9515	0.0182	0.0093	0.0210	0.0588	0.9127
26	-0.05397	0.0198	0.0106	0.0237	0.06476	-0.09577	0.03001	0.00100	-0.04	0.03513	0.9481	0.0183	0.0104	0.0232	0.0601	0.9093
27	-0.05784	0.0198	0.0118	0.0262	0.06733	-0.10068	0.03235	0.00100	-0.04	0.03596	0.9444	0.0183	0.0116	0.0257	0.0622	0.9048
28	-0.06194	0.0197	0.0133	0.0289	0.07094	-0.10679	0.03484	0.00100	-0.04	0.03710	0.9406	0.0182	0.0130	0.0283	0.0652	0.8994
29	-0.06629	0.0195	0.0149	0.0319	0.07574	-0.11423	0.03749	0.00100	-0.04	0.03868	0.9365	0.0179	0.0144	0.0312	0.0692	0.8927
30	-0.07092	0.0192	0.0166	0.0351	0.08182	-0.12312	0.04030	0.00100	-0.04	0.03995	0.9323	0.0174	0.0161	0.0342	0.0743	0.8849
31	-0.07585	0.0187	0.0185	0.0386	0.08934	-0.13359	0.04325	0.00100	-0.04	0.04055	0.9277	0.0169	0.0179	0.0375	0.0805	0.8757
32	-0.08113	0.0181	0.0207	0.0424	0.09842	-0.14573	0.04631	0.00100	-0.04	0.04169	0.9229	0.0162	0.0198	0.0411	0.0879	0.8652
33	-0.08679	0.0173	0.0230	0.0465	0.10912	-0.15963	0.04950	0.00100	-0.05	0.04516	0.9177	0.0153	0.0219	0.0450	0.0965	0.8533
34	-0.09287	0.0163	0.0256	0.0509	0.12147	-0.17529	0.05281	0.00100	-0.05	0.04795	0.9122	0.0143	0.0243	0.0493	0.1063	0.8401
35	-0.09937	0.0150	0.0286	0.0558	0.13550	-0.19269	0.05620	0.00100	-0.05	0.05265	0.9063	0.0130	0.0269	0.0538	0.1172	0.8256
36	-0.10631	0.0134	0.0319	0.0609	0.15135	-0.21195	0.05960	0.00100	-0.06	0.06079	0.9000	0.0115	0.0297	0.0588	0.1292	0.8099
37	-0.11373	0.0114	0.0358	0.0665	0.16945	-0.23340	0.06295	0.00100	-0.07	0.06839	0.8933	0.0096	0.0330	0.0641	0.1426	0.7926
38	-0.12178	0.0089	0.0403	0.0726	0.19042	-0.25763	0.06621	0.00100	-0.08	0.08177	0.8861	0.0074	0.0366	0.0699	0.1577	0.7736

Matrix Exp(D)

	State A	State B	State C	Dead
State A	0.9971	0.0000	0.0000	0.0000
State B	0.0000	0.9434	0.0000	0.0000
State C	0.0000	0.0000	0.9990	0.0000
Dead	0.0000	0.0000	0.0000	1.0000

Matrix P(1) = U * Exp(D) * U^(-1)

	State A	State B	State C	Dead
State A	0.9941	0.0029	0.0006	0.0023
State B	0.0516	0.9464	0.0010	0.0010
State C	0.0000	0.0000	0.9990	0.0010
Dead	0.0000	0.0000	0.0000	1.0000

Intermediate variables

ate B to ...		From State C to Dead		State transition model (Markov trace)				Check	U=						
State C	Dead	State C	Dead	State A	State B	State C	Dead		m	n	o	p	q	r	s
				1.000	0.000	0.000		1.00							
0.0010	0.0010	0.9990	0.0010	0.994	0.003	0.001	0.002329516	1.00	0.0060	0.0000	-0.0002	0.0060951	-0.1046059	0.0000	-0.0002
0.0018	0.0010	0.9972	0.0028	0.987	0.006	0.001	0.005015201	1.00	0.0074	-0.0001	-0.0002	0.0074574	-0.1135968	-0.0001	-0.0002
0.0024	0.0011	0.9965	0.0035	0.980	0.010	0.002	0.008024343	1.00	0.0088	-0.0001	-0.0003	0.0088261	-0.1195943	-0.0001	-0.0003
0.0030	0.0011	0.9956	0.0044	0.971	0.014	0.003	0.011336107	1.00	0.0101	-0.0001	-0.0003	0.0101647	-0.1239675	-0.0001	-0.0003
0.0037	0.0011	0.9947	0.0053	0.962	0.019	0.005	0.014943675	1.00	0.0114	-0.0001	-0.0004	0.0114742	-0.1276544	-0.0001	-0.0004
0.0045	0.0011	0.9939	0.0061	0.952	0.023	0.006	0.018855907	1.00	0.0128	-0.0001	-0.0004	0.0127783	-0.1310971	-0.0001	-0.0004
0.0053	0.0011	0.9930	0.0070	0.941	0.028	0.008	0.023097331	1.00	0.0142	-0.0001	-0.0005	0.0141093	-0.1344783	-0.0001	-0.0005
0.0062	0.0011	0.9921	0.0079	0.930	0.033	0.010	0.027697964	1.00	0.0156	-0.0002	-0.0006	0.0154866	-0.1378468	-0.0002	-0.0006
0.0071	0.0012	0.9905	0.0095	0.917	0.038	0.012	0.03268983	1.00	0.0171	-0.0002	-0.0006	0.0169013	-0.1410031	-0.0002	-0.0006
0.0079	0.0012	0.9888	0.0112	0.904	0.043	0.014	0.038099636	1.00	0.0186	-0.0002	-0.0007	0.0183275	-0.1435779	-0.0002	-0.0007
0.0088	0.0012	0.9878	0.0122	0.891	0.049	0.017	0.043935395	1.00	0.0201	-0.0003	-0.0008	0.0197581	-0.1452255	-0.0002	-0.0008
0.0096	0.0013	0.9862	0.0138	0.876	0.054	0.019	0.050213742	1.00	0.0216	-0.0003	-0.0009	0.0212044	-0.1457376	-0.0003	-0.0009
0.0104	0.0013	0.9856	0.0144	0.861	0.059	0.022	0.056925242	1.00	0.0231	-0.0003	-0.0010	0.0226694	-0.1450618	-0.0003	-0.0010
0.0113	0.0013	0.9853	0.0147	0.846	0.065	0.026	0.064077185	1.00	0.0246	-0.0004	-0.0011	0.0241575	-0.1433309	-0.0003	-0.0011
0.0121	0.0013	0.9849	0.0151	0.829	0.070	0.029	0.071699085	1.00	0.0261	-0.0004	-0.0012	0.0256699	-0.1407819	-0.0004	-0.0012
0.0130	0.0014	0.9834	0.0166	0.812	0.075	0.033	0.079858543	1.00	0.0276	-0.0005	-0.0013	0.0272014	-0.1377628	-0.0004	-0.0013
0.0140	0.0014	0.9815	0.0185	0.793	0.080	0.038	0.088614177	1.00	0.0291	-0.0005	-0.0014	0.0287335	-0.1345599	-0.0005	-0.0014
0.0151	0.0015	0.9801	0.0199	0.774	0.086	0.042	0.09800792	1.00	0.0306	-0.0005	-0.0016	0.0302586	-0.1314934	-0.0005	-0.0016
0.0163	0.0015	0.9777	0.0223	0.755	0.091	0.047	0.108149755	1.00	0.0321	-0.0006	-0.0017	0.0317933	-0.1287699	-0.0005	-0.0017
0.0177	0.0016	0.9750	0.0250	0.734	0.096	0.052	0.119149098	1.00	0.0336	-0.0006	-0.0019	0.0333265	-0.1264127	-0.0006	-0.0019
0.0192	0.0017	0.9721	0.0279	0.712	0.100	0.057	0.131114299	1.00	0.0350	-0.0007	-0.0021	0.0348407	-0.1246341	-0.0006	-0.0021
0.0208	0.0018	0.9697	0.0303	0.690	0.104	0.062	0.144098874	1.00	0.0363	-0.0008	-0.0023	0.0363123	-0.1235807	-0.0007	-0.0023
0.0226	0.0018	0.9683	0.0317	0.667	0.108	0.068	0.158107479	1.00	0.0375	-0.0009	-0.0026	0.0377097	-0.1230829	-0.0008	-0.0026
0.0244	0.0019	0.9671	0.0329	0.643	0.111	0.074	0.173189269	1.00	0.0385	-0.0010	-0.0030	0.0389767	-0.1229627	-0.0009	-0.0030
0.0264	0.0021	0.9662	0.0338	0.618	0.113	0.080	0.189391764	1.00	0.0391	-0.0011	-0.0034	0.0400952	-0.1232808	-0.0010	-0.0034
0.0284	0.0022	0.9655	0.0345	0.593	0.114	0.087	0.206753385	1.00	0.0395	-0.0012	-0.0039	0.0410615	-0.1246689	-0.0012	-0.0039
0.0306	0.0023	0.9647	0.0353	0.567	0.114	0.094	0.225299715	1.00	0.0396	-0.0014	-0.0045	0.041851	-0.1275332	-0.0015	-0.0045
0.0329	0.0025	0.9636	0.0364	0.541	0.113	0.102	0.245060703	1.00	0.0395	-0.0016	-0.0052	0.0424148	-0.1321054	-0.0018	-0.0052
0.0353	0.0028	0.9621	0.0379	0.514	0.110	0.110	0.266076986	1.00	0.0391	-0.0019	-0.0061	0.0427113	-0.1385879	-0.0022	-0.0061
0.0378	0.0031	0.9608	0.0392	0.487	0.106	0.118	0.288307788	1.00	0.0384	-0.0022	-0.0072	0.0427089	-0.1471155	-0.0026	-0.0072
0.0404	0.0034	0.9603	0.0397	0.461	0.101	0.126	0.311651875	1.00	0.0374	-0.0025	-0.0085	0.0423814	-0.1578542	-0.0032	-0.0085
0.0431	0.0038	0.9592	0.0408	0.434	0.095	0.135	0.336140893	1.00	0.0362	-0.0030	-0.0100	0.0416914	-0.1708982	-0.0039	-0.0100
0.0459	0.0043	0.9558	0.0442	0.408	0.088	0.143	0.362046	1.00	0.0346	-0.0035	-0.0120	0.0405644	-0.1862354	-0.0046	-0.0120
0.0487	0.0049	0.9532	0.0468	0.381	0.080	0.150	0.389227421	1.00	0.0326	-0.0041	-0.0143	0.0388937	-0.2037247	-0.0055	-0.0143
0.0515	0.0057	0.9487	0.0513	0.355	0.071	0.157	0.417888656	1.00	0.0301	-0.0048	-0.0171	0.0365237	-0.2231651	-0.0065	-0.0171
0.0542	0.0068	0.9410	0.0590	0.328	0.061	0.162	0.448452711	1.00	0.0269	-0.0056	-0.0205	0.033248	-0.2445254	-0.0075	-0.0205
0.0569	0.0079	0.9339	0.0661	0.302	0.052	0.165	0.480678836	1.00	0.0228	-0.0066	-0.0246	0.0288315	-0.2681583	-0.0089	-0.0246
0.0593	0.0095	0.9215	0.0785	0.276	0.042	0.167	0.515282841	1.00	0.0178	-0.0077	-0.0297	0.0230457	-0.2947543	-0.0103	-0.0297

		Uinverse											D					
u	v	aa	bb	cc	dd	ff	gg	hh	ii	jj	kk	ll	k	l	-j	expk	expl	**exp_j**
-0.0001	-0.0002	157.7162	9.0333371	-56.652086	-110.09742	9.1897335	-9.0333371	0.0578589	-0.2142553	-9322.6497	9322.6497	-5921.5878	-0.0028991	-0.0582497	-0.0010247	0.9971051	0.9434144	0.9989758
0.0000	-0.0002	127.2333	8.2607656	-171.40237	35.908322	8.3525837	-8.2607656	0.1380277	-0.2298457	-24573.614	24573.614	-4543.3596	-0.0034408	-0.0639679	-0.0027758	0.9965651	0.9380351	0.9972281
0.0000	-0.0003	106.4286	7.7869271	-267.52357	153.30799	7.8544551	-7.7869271	0.1782726	-0.2458006	-34690.673	34690.673	-3714.4984	-0.0039498	-0.0681599	-0.0035039	0.996058	0.9341111	0.9965022
0.0000	-0.0003	91.4832	7.455329	-2697.2575	2598.3189	7.501172	-7.455329	0.2167499	-0.2625929	-322662.96	322662.96	-3141.8114	-0.0044505	-0.0715167	-0.0044044	0.9955593	0.9309808	0.9956053
0.0000	-0.0004	80.1600	7.1875903	399.95985	-487.30747	7.2052001	-7.1875903	0.2616258	-0.2792356	44303.03	-44303.03	-2702.4944	-0.0049649	-0.0745292	-0.0052909	0.9950474	0.9281804	0.9947231
0.0000	-0.0004	71.1785	6.950458	226.12967	-304.25866	6.937919	-6.950458	0.3083801	-0.2958411	23202.289	-23202.289	-2342.7054	-0.0055114	-0.0774492	-0.0061156	0.9945037	0.9254741	0.993903
0.0001	-0.0005	63.7660	6.7300364	154.97402	-225.47007	6.690255	-6.7300364	0.3533196	-0.3135383	14775.552	-14775.552	-2037.1131	-0.0061056	-0.0803995	-0.0070281	0.993913	0.9227477	0.9929966
0.0001	-0.0006	57.5014	6.5217368	122.60249	-186.62567	6.4600808	-6.5217368	0.393707	-0.332051	10899.973	-10899.973	-1775.4601	-0.0067519	-0.0834186	-0.0079678	0.9932708	0.919966	0.9920638
0.0002	-0.0006	52.1848	6.3329491	75.359665	-133.8774	6.2551023	-6.3329491	0.4327194	-0.3548726	6318.4436	-6318.4436	-1554.3053	-0.0074466	-0.0863987	-0.0095048	0.9925811	0.9172284	0.9905402
0.0002	-0.0007	47.6877	6.1764466	53.137977	-107.00216	6.087266	-6.1764466	0.4708059	-0.3816253	4225.6894	-4225.6894	-1370.0346	-0.0081883	-0.0891409	-0.0112255	0.9918452	0.9147166	0.9888373
0.0003	-0.0008	43.8468	6.0612099	50.455443	-100.36346	5.9654044	-6.0612099	0.5043448	-0.4085393	3791.3625	-3791.3625	-1218.6221	-0.0089719	-0.0914636	-0.0123038	0.9910683	0.9125945	0.9877716
0.0003	-0.0009	40.4916	5.9901056	42.776557	-89.2583	5.8914122	-5.9901056	0.5425571	-0.4438638	3073.1545	-3073.1545	-1094.7649	-0.009794	-0.093265	-0.0138937	0.9902538	0.9109521	0.9862024
0.0003	-0.0010	37.5198	5.9619207	47.339584	-90.821326	5.8633735	-5.9619207	0.5776208	-0.4790736	3231.69	-3231.69	-992.91214	-0.0106552	-0.0945208	-0.0145233	0.9894014	0.9098088	0.9855817
0.0003	-0.0011	34.8564	5.9705592	59.209381	-100.03632	5.8748281	-5.9705592	0.614809	-0.5190779	3843.1674	-3843.1674	-906.93277	-0.0115684	-0.0953127	-0.0148003	0.9884982	0.9090886	0.9853087
0.0002	-0.0012	32.4547	6.0077425	75.126807	-113.58922	5.9177285	-6.0077425	0.6583736	-0.5683596	4658.8909	-4658.8909	-831.9152	-0.0125505	-0.0957765	-0.0152149	0.9875279	0.9086671	0.9849003
0.0002	-0.0013	30.2887	6.0619198	67.102753	-103.45335	5.9805317	-6.0619198	0.7168607	-0.6354727	4036.5611	-4036.5611	-764.60345	-0.0136105	-0.0960926	-0.0167321	0.9864817	0.9083799	0.9834071
0.0003	-0.0014	28.3398	6.1239473	56.772521	-91.236312	6.0515992	-6.1239473	0.7882991	-0.715951	3326.6249	-3326.6249	-702.75049	-0.0147602	-0.0964068	-0.0186249	0.9853482	0.9080945	0.9815475
0.0003	-0.0016	26.5871	6.1823003	56.267089	-89.036472	6.118093	-6.1823003	0.8656968	-0.8014896	3182.5409	-3182.5409	-643.98273	-0.016025	-0.096901	-0.0201172	0.9841027	0.9076458	0.9800838
0.0004	-0.0017	24.9989	6.2280756	47.484226	-78.711173	6.1722266	-6.2280756	0.9622997	-0.9064506	2608.7596	-2608.7596	-586.91627	-0.0174361	-0.0977178	-0.0225347	0.982715	0.9069048	0.9777173
0.0005	-0.0019	23.5771	6.260206	40.828119	-70.665405	6.2156819	-6.260206	1.0715002	-1.0269762	2172.3146	-2172.3146	-531.32094	-0.0190304	-0.0988999	-0.0252836	0.9811496	0.9058333	0.9750334
0.0005	-0.0021	22.3335	6.2705819	36.194715	-64.798764	6.2431863	-6.2705819	1.186536	-1.1591404	1854.4598	-1854.4598	-477.51641	-0.0208246	-0.1005621	-0.0282854	0.9793907	0.904329	0.9721109
0.0006	-0.0023	21.2758	6.2541813	36.199214	-63.729242	6.2515843	-6.2541813	1.2862253	-1.2836283	1754.0196	-1754.0196	-426.23623	-0.0228278	-0.1027744	-0.0307427	0.9774307	0.9023306	0.9697251
0.0005	-0.0026	20.4048	6.2191955	42.686978	-69.310931	6.2515312	-6.2191955	1.3556988	-1.3880345	1910.5615	-1910.5615	-378.6789	-0.0250449	-0.1054411	-0.0321903	0.9752661	0.8999275	0.9683223
0.0004	-0.0030	19.7361	6.1751471	55.061237	-80.972434	6.2559339	-6.1751471	1.407085	-1.4878718	2247.7086	-2247.7086	-335.06606	-0.0275115	-0.1084813	-0.0334403	0.9728635	0.8971957	0.9671127
0.0003	-0.0034	19.2786	6.1208466	86.918079	-112.31751	6.2700711	-6.1208466	1.429615	-1.5788395	3179.7114	-3179.7114	-294.667	-0.030303	-0.111991	-0.0343538	0.9701516	0.8940523	0.9662296
0.0001	-0.0039	19.0329	6.0338969	225.70901	-250.77586	6.2687748	-6.0338969	1.4159046	-1.6507825	7280.747	-7280.747	-257.15041	-0.0334369	-0.116302	-0.0351289	0.967116	0.8902063	0.965481
-0.0001	-0.0045	18.9949	5.9037373	-438.61962	413.72101	6.2333154	-5.9037373	1.3688423	-1.6984204	-12318.949	12318.949	-222.79988	-0.03691	-0.1216021	-0.0359621	0.9637629	0.8855006	0.9646768
-0.0003	-0.0052	19.1685	5.7299972	-124.85505	99.956571	6.1543823	-5.7299972	1.2978052	-1.7221904	-3025.4233	3025.4233	-191.80749	-0.0407331	-0.1279932	-0.0370968	0.9600853	0.8798594	0.9635829
-0.0006	-0.0061	19.5617	5.5157454	-79.454841	54.377422	6.0286956	-5.5157454	1.209353	-1.7223033	-1647.8397	1647.8397	-164.12494	-0.0449374	-0.1355869	-0.0386754	0.9560574	0.8732032	0.9620629
-0.0010	-0.0072	20.1848	5.2680291	-56.99679	31.543935	5.859825	-5.2680291	1.0997098	-1.6915057	-995.69513	995.69513	-139.6505	-0.0495636	-0.1444757	-0.0399548	0.9516447	0.8654759	0.9608329
-0.0016	-0.0085	21.0555	4.9941184	-42.973506	16.923908	5.6530662	-4.9941184	0.973184	-1.6321318	-620.44733	620.44733	-118.20171	-0.0546598	-0.1547776	-0.0405499	0.9468072	0.8566057	0.9602612
-0.0023	-0.0100	22.2091	4.7038988	-36.396964	9.4840094	5.4180014	-4.7038988	0.8479795	-1.5620822	-430.67176	430.67176	-99.582748	-0.0602834	-0.1665782	-0.041691	0.9414977	0.8465566	0.9591661
-0.0029	-0.0120	23.7228	4.409176	-35.789669	7.6577284	5.1671125	-4.409176	0.737468	-1.4954045	-347.57646	347.57646	-83.569729	-0.066511	-0.1799109	-0.04516	0.9356526	0.8353446	0.9558445
-0.0037	-0.0143	25.7456	4.1216983	-34.45609	4.5888197	4.9151671	-4.1216983	0.624747	-1.4182159	-267.48958	267.48958	-69.939349	-0.073424	-0.1947333	-0.0479541	0.9292067	0.8230542	0.9531776
-0.0045	-0.0171	28.5715	3.8507638	-36.295872	3.8736229	4.6760698	-3.8507638	0.522691	-1.3479969	-221.94192	221.94192	-58.442453	-0.0811112	-0.2109555	-0.0526493	0.9220912	0.8098101	0.9487126
-0.0048	-0.0205	32.7768	3.6000555	-43.645051	7.268233	4.4566462	-3.6000555	0.4304017	-1.2869925	-206.22563	206.22563	-48.780359	-0.0896869	-0.2285736	-0.0607868	0.9142174	0.7956677	0.9410239
-0.0055	-0.0246	39.5789	3.3671189	-52.663004	9.7170054	4.2553917	-3.3671189	0.3324208	-1.2206936	-180.23213	180.23213	-40.629693	-0.0993187	-0.2478137	-0.0683945	0.9054541	0.7805054	0.933892
-0.0053	-0.0297	52.0007	3.146634	-80.803869	25.656576	4.0657234	-3.146634	0.2382021	-1.1572915	-187.32465	187.32465	-33.698286	-0.1102535	-0.2691534	-0.0817654	0.8956071	0.764026	0.9214881
