# Supplementary material for: A Procedure for Deriving Formulas to Convert Transition Rates to Probabilities for Multistate Markov Models
Source: Med Decis Making. 2017 Apr 5;37(7):779–89. doi: 10.1177/0272989X17696997 (PMC5582645; doi:10.1177/0272989X17696997)
Supplement: Supplementary material [file Model_4,_redesigned_for_PDF.rjf_online_supp.pdf]

Supplementary file to accompany Jones, Epstein, Garcia-Mochon "A procedure for deriving formulas to convert transition rates to probabilities for multi-state Markov models"

Model 4. Five-state model with forward transitions only and two death states

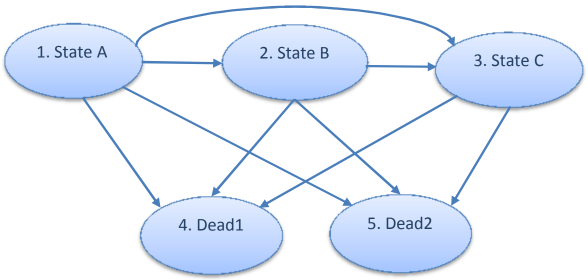

Matrix Q

	State A	State B	State C	Dead1	Dead2
State A	-0.008393	0.0038282	0.0020000	0.0008231	0.0021989
State B	0	-0.1130317	0.0200000	0.0123295	0.0807022
State C	0	0	-0.0030247	0.002	0.00102468
Dead1	0	0	0	0	0
Dead2	0	0	0	0	0

Matrix D

	State A	State B	State C	Dead1	Dead2
State A	-0.0083927	0	0	0	0
State B	0	-0.1130317	0	0	0
State C	0	0	-0.0030247	0	0
Dead1	0	0	0	0	0
Dead2	0	0	0	0	0

Matrix Exp(

State A
State B
State C
Dead1
Dead2

Matrix U

	State A	State B	State C	Dead1	Dead2
State A	1.0000	0.0038	0.0003	0.0000	0.0000
State B	0.0000	-0.1046	0.0001	0.0000	0.0000
State C	0.0000	0.0000	0.0006	0.0000	0.0000
Dead1	0.0000	0.0000	0.0000	0.0000	0.0000
Dead2	0.0000	0.0000	0.0000	0.0000	0.0000

Matrix U^(-1)

	State A	State B	State C	Dead1	Dead2
State A	1.0000	0.0366	-0.5089	0.0396	-0.5673
State B	0	-9.5567	1.7375	1.0117	6.8075
State C	0	0	1693.4293	-1119.74	-573.69
Dead1	0	0	0	-348513.51	0
Dead2	0	0	0	0	-348513.51

Matrix P(1)

State A
State B
State C
Dead1
Dead2

Transition rates

Cycle	From State A to ...					From State B to ...					From State C to ...				
	State A	State B	State C	Dead1	Dead2	State A	State B	State C	Dead1	Dead2	State A	State B	State C	Dead1	Dead2
	a	b	c	d	f		g	h	i	j			k	l	m
0															
1	-0.00839	0.00383	0.002	0.00023	0.00233	0	-0.11303	0.02	0.01233	0.0807022	0	0	-0.00302468	0.002	0.00102468
2	-0.00847	0.00345	0.002	0.00082	0.0022	0	-0.03271	0.02	0.0127	0.00001	0	0	-0.00690323	0.00368009	0.00322314
3	-0.00996	0.00424	0.002	0.00102	0.0027	0	-0.03305	0.02	0.01304	0.00001	0	0	-0.01407576	0.00963005	0.00444571
4	-0.01116	0.00488	0.002	0.00116	0.00313	0	-0.03337	0.02	0.01336	0.00001	0	0	-0.02167902	0.01586172	0.0058173
5	-0.01243	0.00556	0.002	0.00135	0.00352	0	-0.0337	0.02	0.01369	0.00001	0	0	-0.03244381	0.02459459	0.00784922
6	-0.0136	0.0062	0.002	0.00147	0.00393	0	-0.03403	0.02	0.01402	0.00001	0	0	-0.03922099	0.0302809	0.00894009
7	-0.01505	0.00707	0.002	0.00158	0.0044	0	-0.0344	0.02	0.01439	0.00001	0	0	-0.04371028	0.03441821	0.00929207
8	-0.01642	0.00795	0.002	0.00171	0.00476	0	-0.03481	0.02	0.0148	0.00001	0	0	-0.04568207	0.03631584	0.00936623
9	-0.01792	0.0089	0.002	0.00191	0.00511	0	-0.03526	0.02	0.01525	0.00001	0	0	-0.04722041	0.03780198	0.00941843
10	-0.01974	0.00986	0.002	0.00224	0.00564	0	-0.03573	0.02	0.01572	0.00001	0	0	-0.0481752	0.03886429	0.00931091
11	-0.02093	0.01048	0.002	0.00249	0.00595	0	-0.03624	0.02	0.01623	0.00001	0	0	-0.04815448	0.03901385	0.00914063
12	-0.02203	0.01109	0.002	0.00275	0.00619	0	-0.03676	0.02	0.01675	0.00001	0	0	-0.04860241	0.0383752	0.01022721
13	-0.02332	0.01168	0.002	0.00313	0.0065	0	-0.0373	0.02	0.01729	0.00001	0	0	-0.04868938	0.03698403	0.01170535
14	-0.02489	0.01245	0.002	0.00348	0.00695	0	-0.03856	0.02	0.01787	0.0006879	0	0	-0.04986761	0.03770012	0.01216749
15	-0.02709	0.0135	0.002	0.00384	0.00775	0	-0.04175	0.02	0.0185	0.0032498	0	0	-0.05408485	0.04072458	0.01336027
16	-0.02959	0.01462	0.002	0.00417	0.0088	0	-0.04574	0.02	0.01919	0.0065526	0	0	-0.06081391	0.04620648	0.01460743
17	-0.0323	0.01586	0.002	0.00448	0.00996	0	-0.05134	0.02	0.01995	0.0113909	0	0	-0.06447273	0.04914619	0.01532654
18	-0.03535	0.01719	0.002	0.00492	0.01123	0	-0.05987	0.02	0.02081	0.0190564	0	0	-0.06486528	0.04934194	0.01552334
19	-0.03776	0.01799	0.002	0.00545	0.01232	0	-0.06881	0.02	0.02179	0.0270242	0	0	-0.06237906	0.04696694	0.01541212
20	-0.03946	0.01839	0.002	0.00607	0.01301	0	-0.07658	0.02	0.02291	0.0336674	0	0	-0.05902808	0.04350162	0.01552646
21	-0.04099	0.01876	0.002	0.00674	0.01348	0	-0.08294	0.02	0.0242	0.0387371	0	0	-0.0556846	0.04001171	0.01567289
22	-0.04264	0.01918	0.002	0.00740	0.01406	0	-0.08906	0.02	0.02565	0.0434043	0	0	-0.05343443	0.03713233	0.0163021
23	-0.04509	0.01959	0.002	0.00818	0.01531	0	-0.09318	0.02	0.02727	0.0459115	0	0	-0.05208771	0.03415617	0.01793154
24	-0.04736	0.02014	0.002	0.00884	0.01639	0	-0.09747	0.02	0.02906	0.0484072	0	0	-0.05155511	0.03250439	0.01905072
25	-0.04998	0.02074	0.002	0.00941	0.01783	0	-0.1059	0.02	0.03108	0.0548212	0	0	-0.05298998	0.03287364	0.02011634
26	-0.05289	0.02134	0.002	0.01009	0.01946	0	-0.11322	0.02	0.03332	0.0599011	0	0	-0.05949255	0.03584583	0.02364672
27	-0.05756	0.02243	0.002	0.01094	0.02219	0	-0.12057	0.02	0.03575	0.064821	0	0	-0.06598461	0.03948374	0.02650087
28	-0.06215	0.02356	0.002	0.01161	0.02498	0	-0.12727	0.02	0.03838	0.0688914	0	0	-0.07561228	0.04474291	0.03086937
29	-0.06852	0.02474	0.002	0.01258	0.0292	0	-0.13685	0.02	0.04121	0.0756371	0	0	-0.08408207	0.0497538	0.03432827
30	-0.07399	0.02501	0.002	0.01364	0.03334	0	-0.15202	0.02	0.04426	0.0877552	0	0	-0.09449063	0.05647766	0.03801297
31	-0.07954	0.02503	0.002	0.01531	0.0372	0	-0.17746	0.02	0.04755	0.1099135	0	0	-0.10406749	0.06250911	0.04155838
32	-0.08489	0.0249	0.002	0.01724	0.04075	0	-0.19691	0.02	0.0511	0.1258187	0	0	-0.11224931	0.06811356	0.04413575
33	-0.09156	0.02388	0.002	0.02008	0.0456	0	-0.21132	0.02	0.05492	0.1363978	0	0	-0.12473968	0.07732715	0.04741253
34	-0.10078	0.02175	0.002	0.0254	0.05162	0	-0.22009	0.02	0.05904	0.1410489	0	0	-0.1642407	0.11095113	0.05328957
35	-0.10464	0.01921	0.002	0.02863	0.05479	0	-0.22226	0.02	0.06347	0.1387927	0	0	-0.2053823	0.15038301	0.05499929
36	-0.10315	0.0158	0.002	0.0305	0.05485	0	-0.21869	0.02	0.06819	0.1304978	0	0	-0.23089352	0.17757123	0.05332229
37	-0.09733	0.01225	0.002	0.03099	0.05208	0	-0.21028	0.02	0.07323	0.1170471	0	0	-0.24474989	0.19494812	0.04980177
38	-0.08861	0.00898	0.002	0.03014	0.04749	0	-0.20351	0.02	0.07863	0.1048802	0	0	-0.24458845	0.20024507	0.04434338
39	-0.0767	0.00591	0.002	0.02809	0.0407	0	-0.36573	0.02	0.24085	0.1048802	0	0	-0.23493304	0.19650454	0.0384285

Transition probabilitie

Cycle	From State A to ...					From State B to		
	State A	State B	State C	Dead1	Dead2	State A	State B	State C
0								
1	0.9916	0.0036	0.0020	0.0003	0.0025	0	0.8931	0.0189
2	0.9916	0.0034	0.0020	0.0008	0.0022	0	0.9678	0.0196
3	0.9901	0.0041	0.0020	0.0010	0.0027	0	0.9675	0.0195
4	0.9889	0.0048	0.0020	0.0012	0.0031	0	0.9672	0.0195
5	0.9876	0.0054	0.0020	0.0014	0.0035	0	0.9669	0.0193
6	0.9865	0.0061	0.0020	0.0015	0.0039	0	0.9665	0.0193
7	0.9851	0.0069	0.0020	0.0017	0.0044	0	0.9662	0.0192
8	0.9837	0.0077	0.0020	0.0018	0.0047	0	0.9658	0.0192
9	0.9822	0.0087	0.0020	0.0020	0.0051	0	0.9654	0.0192
10	0.9805	0.0096	0.0020	0.0023	0.0056	0	0.9649	0.0192
11	0.9793	0.0102	0.0020	0.0026	0.0059	0	0.9644	0.0192
12	0.9782	0.0108	0.0020	0.0028	0.0061	0	0.9639	0.0192
13	0.9770	0.0113	0.0020	0.0032	0.0064	0	0.9634	0.0192
14	0.9754	0.0121	0.0020	0.0036	0.0069	0	0.9622	0.0191
15	0.9733	0.0130	0.0021	0.0040	0.0077	0	0.9591	0.0191
16	0.9708	0.0141	0.0021	0.0043	0.0087	0	0.9553	0.0190
17	0.9682	0.0152	0.0021	0.0046	0.0099	0	0.9500	0.0189
18	0.9653	0.0164	0.0021	0.0051	0.0112	0	0.9419	0.0188
19	0.9629	0.0171	0.0021	0.0056	0.0123	0	0.9335	0.0187
20	0.9613	0.0174	0.0021	0.0062	0.0131	0	0.9263	0.0187
21	0.9598	0.0176	0.0021	0.0069	0.0136	0	0.9204	0.0187
22	0.9583	0.0180	0.0021	0.0075	0.0142	0	0.9148	0.0186
23	0.9559	0.0183	0.0021	0.0083	0.0154	0	0.9110	0.0186
24	0.9537	0.0187	0.0021	0.0089	0.0165	0	0.9071	0.0186
25	0.9512	0.0192	0.0021	0.0095	0.0180	0	0.8995	0.0185
26	0.9485	0.0196	0.0021	0.0102	0.0196	0	0.8930	0.0183
27	0.9441	0.0205	0.0021	0.0111	0.0223	0	0.8864	0.0182
28	0.9397	0.0214	0.0021	0.0117	0.0250	0	0.8805	0.0181
29	0.9338	0.0223	0.0021	0.0127	0.0291	0	0.8721	0.0179
30	0.9287	0.0223	0.0021	0.0137	0.0332	0	0.8590	0.0177
31	0.9235	0.0220	0.0020	0.0153	0.0371	0	0.8374	0.0174
32	0.9186	0.0216	0.0020	0.0172	0.0405	0	0.8213	0.0171
33	0.9125	0.0205	0.0020	0.0199	0.0451	0	0.8095	0.0169
34	0.9041	0.0185	0.0019	0.0249	0.0505	0	0.8024	0.0165
35	0.9007	0.0163	0.0019	0.0279	0.0533	0	0.8007	0.0162
36	0.9020	0.0135	0.0018	0.0296	0.0531	0	0.8036	0.0160
37	0.9073	0.0105	0.0018	0.0301	0.0503	0	0.8104	0.0159
38	0.9152	0.0078	0.0018	0.0293	0.0459	0	0.8159	0.0160
39	0.9262	0.0048	0.0018	0.0278	0.0395	0	0.6937	0.0148

D)

State A	State B	State C	Dead1	Dead2
0.9916424	0	0	0	0
0	0.8931223	0	0	0
0	0	0.9969799	0	0
0	0	0	1	0
0	0	0	0	1

= U * Exp(D) * U^(-1)

State A	State B	State C	Dead1	Dead2	
0.992	0.004	0.002	0.0003	0.002	1
0	0.893	0.0189	0.0117	0.0763	1
0	0	0.9970	0.002	0.001	1
0	0	0.0	1.0	0.0	1
0	0	0.0	0.0	1.0	1

es

...		From State C to ...				
Dead1	Dead2	State A	State B	State C	Dead1	Dead2
0.0117	0.0763	0	0	0.9970	0.0020	0.0010
0.0125	0.0000	0	0	0.9931	0.0037	0.0032
0.0129	0.0001	0	0	0.9860	0.0096	0.0044
0.0133	0.0001	0	0	0.9786	0.0157	0.0058
0.0137	0.0001	0	0	0.9681	0.0242	0.0077
0.0141	0.0001	0	0	0.9615	0.0297	0.0088
0.0145	0.0001	0	0	0.9572	0.0337	0.0091
0.0149	0.0001	0	0	0.9553	0.0355	0.0092
0.0153	0.0001	0	0	0.9539	0.0369	0.0092
0.0158	0.0001	0	0	0.9530	0.0379	0.0091
0.0163	0.0001	0	0	0.9530	0.0381	0.0089
0.0168	0.0001	0	0	0.9526	0.0375	0.0100
0.0173	0.0001	0	0	0.9525	0.0361	0.0114
0.0179	0.0008	0	0	0.9514	0.0368	0.0119
0.0185	0.0033	0	0	0.9474	0.0396	0.0130
0.0192	0.0065	0	0	0.9410	0.0448	0.0142
0.0199	0.0113	0	0	0.9376	0.0476	0.0148
0.0207	0.0186	0	0	0.9372	0.0478	0.0150
0.0215	0.0263	0	0	0.9395	0.0455	0.0149
0.0225	0.0326	0	0	0.9427	0.0422	0.0151
0.0236	0.0373	0	0	0.9458	0.0389	0.0152
0.0249	0.0417	0	0	0.9480	0.0362	0.0159
0.0264	0.0440	0	0	0.9492	0.0333	0.0175
0.0280	0.0463	0	0	0.9498	0.0317	0.0186
0.0298	0.0522	0	0	0.9484	0.0320	0.0196
0.0318	0.0569	0	0	0.9422	0.0348	0.0230
0.0341	0.0613	0	0	0.9361	0.0382	0.0256
0.0365	0.0650	0	0	0.9272	0.0431	0.0297
0.0390	0.0710	0	0	0.9194	0.0477	0.0329
0.0416	0.0818	0	0	0.9098	0.0539	0.0363
0.0441	0.1011	0	0	0.9012	0.0594	0.0395
0.0470	0.1146	0	0	0.8938	0.0644	0.0417
0.0502	0.1234	0	0	0.8827	0.0727	0.0446
0.0540	0.1271	0	0	0.8485	0.1023	0.0491
0.0582	0.1249	0	0	0.8143	0.1359	0.0497
0.0628	0.1177	0	0	0.7938	0.1586	0.0476
0.0677	0.1060	0	0	0.7829	0.1729	0.0442
0.0729	0.0953	0	0	0.7830	0.1776	0.0393
0.2033	0.0882	0	0	0.7906	0.1751	0.0342

State transition model (Markov trace)

State A	State B	State C	Dead 1	Dead2	Check
1.000	0	0	0	0	1.000
0.992	0.004	0.002	0.000	0.002	1.000
0.983	0.007	0.004	0.001	0.005	1.000
0.974	0.011	0.006	0.002	0.007	1.000
0.963	0.015	0.008	0.004	0.010	1.000
0.951	0.020	0.010	0.005	0.014	1.000
0.938	0.025	0.012	0.008	0.018	1.000
0.924	0.030	0.014	0.010	0.022	1.000
0.909	0.037	0.016	0.012	0.026	1.000
0.893	0.043	0.018	0.015	0.031	1.000
0.875	0.050	0.019	0.019	0.036	1.000
0.857	0.057	0.021	0.023	0.042	1.000
0.839	0.065	0.023	0.027	0.047	1.000
0.819	0.072	0.025	0.031	0.053	1.000
0.799	0.079	0.027	0.037	0.059	1.000
0.778	0.086	0.028	0.042	0.065	1.000
0.755	0.093	0.030	0.049	0.073	1.000
0.731	0.100	0.031	0.055	0.082	1.000
0.706	0.106	0.033	0.063	0.093	1.000
0.680	0.111	0.034	0.070	0.105	1.000
0.653	0.115	0.036	0.078	0.118	1.000
0.627	0.117	0.037	0.087	0.131	1.000
0.601	0.118	0.039	0.096	0.146	1.000
0.574	0.119	0.040	0.105	0.161	1.000
0.548	0.119	0.042	0.115	0.177	1.000
0.521	0.117	0.043	0.125	0.194	1.000
0.494	0.115	0.044	0.136	0.211	1.000
0.467	0.112	0.044	0.147	0.231	1.000
0.438	0.109	0.044	0.158	0.251	1.000
0.409	0.104	0.043	0.170	0.273	1.000
0.380	0.099	0.042	0.182	0.296	1.000
0.351	0.091	0.040	0.195	0.322	1.000
0.323	0.082	0.038	0.208	0.349	1.000
0.294	0.073	0.036	0.221	0.375	1.000
0.266	0.064	0.032	0.236	0.401	1.000
0.240	0.056	0.028	0.252	0.425	1.000
0.216	0.048	0.023	0.267	0.445	1.000
0.196	0.041	0.019	0.281	0.462	1.000
0.180	0.035	0.016	0.293	0.476	1.000
0.166	0.025	0.014	0.308	0.487	1.000

Intermediate variables

U

1	b	n	o	p	q	r	s	u	v	w	x
1											
1	0.004	0.000	-0.000001	-0.000002	-0.105	0.000	0.000	0.000	0.001	-0.000002	-0.000001
1	0.003	0.000	-0.000001	-0.000001	-0.024	0.000	0.000	0.000	0.000	-0.000001	-0.000001
1	0.004	0.000	-0.000003	-0.000002	-0.023	0.000	0.000	0.000	0.000	-0.000003	-0.000001
1	0.005	0.000	-0.000005	-0.000003	-0.022	0.000	0.000	0.000	0.000	-0.000006	-0.000002
1	0.006	0.000	-0.000008	-0.000005	-0.021	0.000	0.000	0.000	0.000	-0.000010	-0.000003
1	0.006	0.000	-0.000011	-0.000007	-0.020	-0.001	0.000	0.000	0.000	-0.000014	-0.000004
1	0.007	0.000	-0.000014	-0.000009	-0.019	-0.001	0.000	0.000	0.000	-0.000018	-0.000005
1	0.008	0.000	-0.000016	-0.000010	-0.018	-0.001	0.000	0.000	0.000	-0.000021	-0.000005
1	0.009	0.000	-0.000019	-0.000011	-0.017	-0.001	0.000	0.000	0.000	-0.000024	-0.000006
1	0.010	0.000	-0.000022	-0.000012	-0.016	-0.001	0.000	0.000	0.000	-0.000027	-0.000007
1	0.010	0.000	-0.000024	-0.000013	-0.015	-0.001	0.000	0.000	0.000	-0.000030	-0.000007
1	0.011	0.000	-0.000025	-0.000014	-0.015	-0.001	0.000	0.000	0.000	-0.000031	-0.000008
1	0.012	0.000	-0.000027	-0.000015	-0.014	-0.001	0.000	0.000	0.000	-0.000032	-0.000010
1	0.012	0.000	-0.000030	-0.000018	-0.014	0.000	0.000	0.000	0.000	-0.000036	-0.000012
1	0.014	0.000	-0.000037	-0.000025	-0.015	-0.001	0.000	0.000	0.000	-0.000046	-0.000015
1	0.015	0.000	-0.000046	-0.000036	-0.016	-0.001	0.000	0.000	0.000	-0.000063	-0.000020
1	0.016	0.000	-0.000056	-0.000051	-0.019	-0.001	0.000	0.000	0.000	-0.000081	-0.000025
1	0.017	0.000	-0.000065	-0.000072	-0.025	-0.001	0.000	0.000	0.000	-0.000104	-0.000033
1	0.018	0.000	-0.000071	-0.000091	-0.031	0.000	0.000	0.000	0.000	-0.000122	-0.000040
1	0.018	0.000	-0.000075	-0.000103	-0.037	0.000	0.000	0.000	0.000	-0.000131	-0.000047
1	0.019	0.000	-0.000078	-0.000111	-0.042	0.000	0.000	0.000	0.000	-0.000136	-0.000053
1	0.019	0.000	-0.000082	-0.000121	-0.046	0.000	0.000	0.000	0.000	-0.000141	-0.000062
1	0.020	0.000	-0.000087	-0.000132	-0.048	0.000	0.000	0.000	0.000	-0.000144	-0.000075
1	0.020	0.000	-0.000094	-0.000144	-0.050	0.000	0.000	0.000	0.000	-0.000150	-0.000088
1	0.021	0.001	-0.000108	-0.000173	-0.056	0.000	0.000	0.000	0.000	-0.000174	-0.000106
1	0.021	0.001	-0.000134	-0.000223	-0.060	0.000	0.000	0.000	0.000	-0.000215	-0.000142
1	0.022	0.001	-0.000167	-0.000291	-0.063	0.000	0.000	0.000	0.000	-0.000274	-0.000184
1	0.024	0.001	-0.000213	-0.000386	-0.065	0.000	0.000	0.000	-0.001	-0.000354	-0.000244
1	0.025	0.001	-0.000269	-0.000520	-0.068	0.000	0.000	0.000	-0.001	-0.000467	-0.000322
1	0.025	0.001	-0.000346	-0.000717	-0.078	0.000	0.000	-0.001	-0.001	-0.000635	-0.000428
1	0.025	0.001	-0.000460	-0.001009	-0.098	0.000	0.000	-0.001	-0.002	-0.000882	-0.000587
1	0.025	0.001	-0.000585	-0.001292	-0.112	-0.001	-0.001	-0.001	-0.002	-0.001139	-0.000738
1	0.024	0.001	-0.000763	-0.001651	-0.120	-0.001	-0.001	-0.002	-0.003	-0.001496	-0.000917
1	0.022	0.001	-0.001226	-0.002417	-0.119	-0.001	-0.001	-0.002	-0.004	-0.002461	-0.001182
1	0.019	0.000	-0.001682	-0.003094	-0.118	-0.002	-0.002	-0.003	-0.002	-0.003497	-0.001279
1	0.016	0.000	-0.001923	-0.003286	-0.116	-0.003	-0.002	-0.003	0.002	-0.004006	-0.001203
1	0.012	0.000	-0.001944	-0.003064	-0.113	-0.003	-0.002	-0.003	0.005	-0.003990	-0.001019
1	0.009	0.000	-0.001790	-0.002620	-0.115	-0.003	-0.002	-0.002	0.006	-0.003611	-0.000800
1	0.006	0.000	-0.002915	-0.003675	-0.289	-0.003	-0.005	-0.002	-0.021	-0.005512	-0.001078

Uinverse

y		aa	bb	cc	dd	ff	gg	hh	ii	jj	kk	ll	mm	**expa**	**expg**	**expk**
	1															
-2.87E-06	1	0.0366	-0.5089	0.0396	-0.5673	-9.5567	1.7375	1.0117	6.8075	1693.4293	-1119.7411	-573.6882	-348513.514	0.9916424	0.8931223	0.9969799
-1.91E-06	1	0.1421	-3.0958	1.0351	0.9186	-41.2544	31.9750	12.4178	-3.1383	24772.6855	-13206.2400	-11566.4455	-523048.024	0.9915682	0.9678217	0.9931205
-4.63E-06	1	0.1836	1.3781	-1.6751	-0.8866	-43.3114	45.6578	3.7825	-6.1289	-12804.3466	8760.2018	4044.1448	-215852.058	0.9900901	0.9674922	0.9860228
-8.08E-06	1	0.2196	0.6077	-1.2301	-0.5972	-45.0251	77.0099	-18.5743	-13.4105	-8131.8853	5949.7933	2182.0920	-123825.612	0.9888995	0.9671784	0.9785543
-1.36E-05	1	0.2613	0.3610	-1.1107	-0.5116	-47.0224	751.3890	-529.3473	-175.0192	-39918.8230	30261.1526	9657.6704	-73597.3967	0.987648	0.9668659	0.9680768
-1.81E-05	1	0.3033	0.3148	-1.1222	-0.4960	-48.9280	-188.6501	188.0086	49.5695	7523.1444	-5808.3078	-1714.8366	-55102.5168	0.9864964	0.9665388	0.9615382
-2.26E-05	1	0.3653	0.3247	-1.1970	-0.4930	-51.6710	-111.0424	132.7071	30.0064	3749.2080	-2952.1894	-797.0185	-44183.3525	0.9850622	0.9661813	0.9572312
-2.61E-05	1	0.4324	0.3639	-1.2984	-0.4979	-54.3847	-100.0661	127.5125	26.9383	3144.4598	-2499.7488	-644.7110	-38284.3759	0.9837094	0.9657867	0.9553456
-2.98E-05	1	0.5134	0.4187	-1.4266	-0.5055	-57.6699	-96.4066	128.3061	25.7704	2852.3369	-2283.4190	-568.9179	-33525.7882	0.9822431	0.9653578	0.9538771
-3.4E-05	1	0.6166	0.5040	-1.5969	-0.5237	-62.5207	-100.5037	136.8193	26.2051	2826.5521	-2280.2592	-546.2930	-29428.7886	0.9804545	0.9648972	0.9529668
-3.65E-05	1	0.6847	0.5764	-1.7246	-0.5366	-65.3187	-109.6178	147.2678	27.6687	3081.8534	-2496.8594	-584.9940	-27383.9932	0.9792901	0.9644118	0.9529866
-3.94E-05	1	0.7530	0.6421	-1.8157	-0.5794	-67.8962	-114.6566	150.6342	31.9186	3177.6272	-2508.9719	-668.6553	-25406.8797	0.9782103	0.9639084	0.9525598
-4.24E-05	1	0.8356	0.7376	-1.9237	-0.6494	-71.5190	-125.6038	157.6885	39.4343	3461.1873	-2629.0878	-832.0995	-23611.8713	0.9769507	0.9633858	0.9524769
-4.78E-05	1	0.9110	0.8094	-2.0202	-0.7003	-73.1539	-129.3380	160.3698	42.1221	3538.6228	-2675.2135	-863.4093	-20899.8703	0.9754213	0.9621782	0.9513554
-6.12E-05	1	0.9216	0.7569	-1.9088	-0.7697	-68.2450	-110.6096	138.1418	40.7128	3002.2219	-2260.6003	-741.6216	-16348.5361	0.9732717	0.9591143	0.9473517
-8.23E-05	1	0.9050	0.6437	-1.7331	-0.8156	-61.9213	-82.1517	108.9657	35.1072	2124.4802	-1614.1825	-510.2977	-12149.8841	0.970844	0.9552912	0.9409983
-0.000107	1	0.8328	0.5798	-1.5354	-0.8772	-52.5035	-79.9750	96.9564	35.5221	2367.0084	-1804.3202	-562.6883	-9353.85215	0.9682195	0.949953	0.9375617
-0.000137	1	0.7012	0.5428	-1.3099	-0.9341	-40.7824	-163.1398	148.6377	55.2845	6775.4741	-5153.9905	-1621.4836	-7285.84195	0.9652721	0.9418911	0.9371937
-0.000162	1	0.5794	0.5519	-1.1651	-0.9662	-32.2062	100.1651	-58.1723	-9.7866	-6316.4207	4755.8099	1560.6108	-6169.96408	0.9629443	0.9335043	0.9395267
-0.000178	1	0.4953	0.6085	-1.1121	-0.9917	-26.9400	30.6980	-9.3778	5.6199	-2911.6431	2145.7786	765.8645	-5606.19706	0.9613082	0.9262791	0.9426803
-0.000189	1	0.4472	0.7446	-1.1555	-1.0363	-23.8360	17.4915	-1.4829	7.8274	-2496.1669	1793.6001	702.5668	-5282.95359	0.9598431	0.9204074	0.9458374
-0.000203	1	0.4131	0.9505	-1.2500	-1.1136	-21.5428	12.0950	1.1624	8.2854	-2600.1260	1806.8638	793.2622	-4928.47482	0.9582582	0.9147933	0.9479681
-0.000219	1	0.4074	1.4503	-1.5265	-1.3313	-20.7957	10.1222	2.3747	8.2988	-3477.9611	2280.6499	1197.3112	-4569.56329	0.9559113	0.9110323	0.9492456
-0.000238	1	0.4019	2.3941	-2.0763	-1.7197	-19.9576	8.6936	3.0514	8.2126	-5194.6514	3275.1162	1919.5352	-4201.76406	0.9537418	0.907131	0.9497513
-0.00028	1	0.3709	3.1277	-2.4761	-2.0225	-17.8821	6.7594	3.1497	7.9730	-6276.4762	3893.7667	2382.7095	-3565.52067	0.9512496	0.8995142	0.9483895
-0.000356	1	0.3538	1.3742	-1.3450	-1.3831	-16.5760	6.1708	2.9239	7.4813	-2818.3601	1698.1363	1120.2238	-2807.18492	0.9484862	0.8929576	0.9422426
-0.000458	1	0.3559	1.0830	-1.1540	-1.2849	-15.8712	5.8150	2.8017	7.2545	-2175.7001	1301.8911	873.8090	-2183.51231	0.9440609	0.8864134	0.9361453
-0.000598	1	0.3617	0.6858	-0.9039	-1.1437	-15.3553	5.9450	2.5405	6.8699	-1437.5964	850.6852	586.9112	-1672.09302	0.9397449	0.8804953	0.9271756
-0.000788	1	0.3621	0.5938	-0.8325	-1.1233	-14.6348	5.5472	2.3903	6.6973	-1217.6023	720.4906	497.1117	-1268.41488	0.9337775	0.8721034	0.9193558
-0.001063	1	0.3206	0.4104	-0.6893	-1.0417	-12.8169	4.4561	2.0762	6.2846	-848.1272	506.9311	341.1961	-940.861193	0.9286772	0.8589747	0.9098363
-0.001469	1	0.2556	0.2900	-0.5732	-0.9725	-10.2123	2.7828	1.7561	5.6734	-555.5269	333.6824	221.8445	-680.743346	0.9235397	0.8373921	0.9011645
-0.001876	1	0.2223	0.2356	-0.5258	-0.9320	-8.9264	2.1086	1.5869	5.2309	-431.6577	261.9325	169.7252	-532.96443	0.9186162	0.8212608	0.8938214
-0.002414	1	0.1994	0.1805	-0.4913	-0.8886	-8.3503	1.9289	1.4644	4.9570	-348.1463	215.8188	132.3276	-414.31395	0.9125028	0.8095149	0.8827267
-0.003643	1	0.1823	0.0890	-0.4569	-0.8144	-8.3812	3.0011	0.7355	4.6445	-282.1228	190.5852	91.5376	-274.500689	0.9041332	0.8024436	0.8485378
-0.004776	1	0.1633	0.0523	-0.4478	-0.7678	-8.5015	10.0732	-4.3879	2.8162	-588.0437	430.5716	157.4721	-209.360783	0.9006532	0.8007058	0.8143359
-0.005208	1	0.1368	0.0371	-0.4499	-0.7239	-8.6547	-14.1851	14.2167	8.6232	641.5077	-493.3587	-148.1491	-191.999364	0.9019942	0.80357	0.793824
-0.005009	1	0.1085	0.0283	-0.4567	-0.6800	-8.8534	-5.1364	7.8452	6.1446	196.7644	-156.7267	-40.0377	-199.644945	0.9072602	0.8103603	0.7829003
-0.004411	1	0.0782	0.0228	-0.4611	-0.6399	-8.7032	-4.2375	7.5321	5.4085	156.0753	-127.7792	-28.2961	-226.720623	0.915202	0.8158612	0.7830267
-0.00659	2	0.0205	0.0152	-0.4694	-0.5662	-3.4599	0.5291	1.9942	0.9366	-48.3180	40.4145	7.9035	-151.744915	0.9261694	0.693692	0.7906238
