# Supplementary material for: A Procedure for Deriving Formulas to Convert Transition Rates to Probabilities for Multistate Markov Models
Source: Med Decis Making. 2017 Apr 5;37(7):779–89. doi: 10.1177/0272989X17696997 (PMC5582645; doi:10.1177/0272989X17696997)
Supplement: Supplementary material [file Procedure_for_transition_probabilities,_cover_sheet_for_supplementary_materials.rjf_online_supp.doc]

A procedure for deriving formulas to convert transition rates to probabilities for multi-state Markov models

The supplementary materials are available at:

<http://dx.doi.org/10.17863/CAM.6025>
